# Biocontrol potential of newly isolated *Streptomyces noursei* D337-11 from disease suppressive soil and its metabolites against *Fusarium oxysporum* f. sp. *cubense* in banana plants

**DOI:** 10.3389/fmicb.2025.1655103

**Published:** 2025-08-26

**Authors:** Dengbo Zhou, Xinxin He, Yufeng Chen, Chunting Li, Wei Wang, Zhiqiang Pan, Yankun Zhao, Yongzan Wei, Junting Feng, Miaoyi Zhang, Dengfeng Qi, Xiaojuan Li, Kai Li, Tao Jing, Jianghui Xie

**Affiliations:** ^1^National Key Laboratory for Tropical Crop Breeding, Sanya Research Institute and Institute of Tropical Bioscience and Biotechnology, Chinese Academy of Tropical Agricultural Sciences, Sanya, China; ^2^Natural Products Utilization Research Unit, Agricultural Research Service, U.S. Department of Agriculture, University of Mississippi, Oxford, MS, United States

**Keywords:** *Streptomyces noursei*, genome-guided differential metabolomics, banana fusarium wilt, naringenin, target protein

## Abstract

Banana *Fusarium* wilt, caused by *Fusarium oxysporum* f. sp. *cubense*, threatens global banana production. Biocontrol using *Streptomyces* is a promising strategy. Here, we identified *Streptomyces noursei* D337-11 from disease suppressive banana soils, which exhibited dual functionality in controlling *F. oxysporum* f. sp. *cubense* tropical race 4 (*Foc* TR4), achieving a 65.35% suppression of disease, and promoting plant growth in pot experiments. The extracts from this strain restored the soil microbiota disrupted by *Foc* TR4, particularly, reviving *Saccharimonadales* populations. Using integrated untargeted metabolomics (LC-ESIMS/MS) and genomic analysis, we identified five bioactive metabolites, including naringenin, a flavonoid linked to a 100% homologous biosynthetic gene cluster (Region 52.1). Genome-guided differential metabolomics established the genetic basis for flavonoid production. Mechanistic characterization indicated that naringenin exhibits antifungal activity via dual-target inhibition: molecular docking showed strong binding to the trypsin of *Foc* TR4 (N1RZA7, −6.6 kcal/mol) and nitroalkane oxidase (N1R9V2, −8.4 kcal/mol), which disrupts cellular integrity as evidenced by ultrastructural anomalies. We provide the first evidence of *Streptomyces*-derived naringenin as a multi-target antifungal agent. Overall, this study provides a theoretical basis for exploring the application of microbial flavonoids in biological control of fungal diseases.

## Graphical abstract

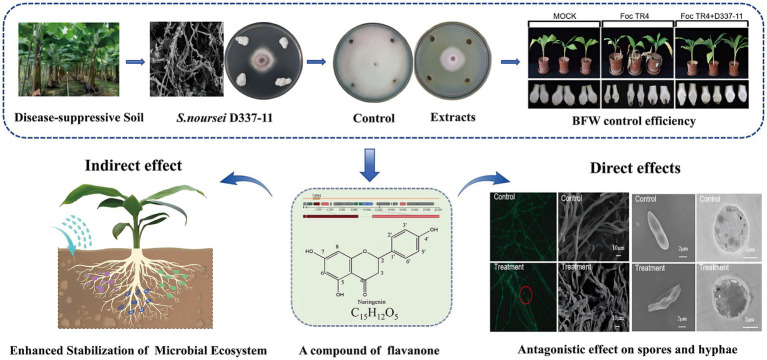

## 1 Introduction

Banana (*Musa* spp.) is a key staple food and fruit in the world. However, the banana industry is facing a severe threat from *Fusarium* wilt disease caused by *Fusarium oxysporum* f. sp. *cubense* tropical race 4 (*Foc* TR4) (Cheng et al., 2024; Chen et al., 2018). This soil-borne pathogen can disrupt the vascular systems of the plants, leading to yield losses exceeding 50% within 2 years of infection (Jing et al., 2020; Tao et al., 2023). Currently, there are no effective chemical fungicides or completely resistant varieties available. This poses a significant challenge to the sustainable development of the banana industry. Biological control agents (BCAs) such as *Streptomyces, Bacillus,* and *Trichoderma* are considered as a promising strategy to suppress the spread of banana fusarium wilt. Particularly, it is urgent to explore microbial-derived antifungal agents from the genus *Streptomyces* (Li et al., 2021; Zou et al., 2021).

The genus *Streptomyces* which belongs to Actinomycetes, are the most widely distributed microbes in nature. Approximately 75% of agricultural antibiotics are derived from *Streptomyces* (Aigle et al., 2014). Despite the current reports on diverse *Streptomyces species* with antagonistic activity against *Fusarium* wilt (Shen et al., 2016; Qi et al., 2021), the discovery of new antagonistic microbial strains and novel active substances has been limited (AbdElgawad et al., 2020; Yun et al., 2021). This limitation arises because the chemical complexity of their metabolomes has hindered the targeted identification of antifungal agents (Spooren et al., 2024). The advances in multi-omics technologies now enable researchers to explore these microbial resources and efficient antifungal substances systematically.

Previous research has established that disease-suppressive soils harbor functional microbes capable of combating soil-borne fungal pathogens (Zhou et al., 2019). Specifically, *Streptomyces* and its antibiotics play an important role in forming a disease-suppressive soil microbial community (Cha et al., 2016; Chen et al., 2024a). Therefore, suppressive soils from banana orchards can serve as valuable sources for isolating highly efficient antifungal strains. In our present study, *Streptomyces noursei* D337-11 was isolated and identified from the disease-suppressive soil in the selected banana orchard. This strain exhibited dual functionality: it suppressed *Foc* TR4 with a disease control efficacy of 65.34% and promoted plant growth. By integrating metabolomics and genomics as well as target compound guided separation, we identified naringenin, a flavonoid compound with previously unreported antifungal activity against *Foc* TR4. While flavonoids are predominantly studied in plants, they exhibit untapped potential in microbial systems for disease management. In addition, we deeply analyzed the antifungal mechanism of this compound, which provided a theoretical basis for exploring the antifungal effects of microbial flavonoids.

## 2 Materials and methods

### 2.1 Isolation of actinomycetes and screening of antagonistic strains

Soil samples of the banana rhizosphere were collected from Six Teams Banana Farm (109°30′1.998″E,19°34′38.644″N, Danzhou, Hainan Province, China). The incidence of banana *Fusarium* wilt on this farm has been controlled and maintained below 0.1% over five consecutive years. Actinomycetes were isolated using a serial dilution method. To prepare soil suspension, 5 g of fresh soil samples were transferred into 45 mL sterile water to create a soil suspension. Soil suspension was diluted from 10^−2^ to 10^−3^ fold. Then 100 μL of the suspension were spread onto starch-casein agar (SCA), glycerol-asparagine (GA), and Gause’s No. 1 media, respectively. Distinct colonies were then purified on yeast extract (YE) agar and stored at −80°C. The antagonistic activity against *Foc* TR4 was evaluated using the spot inoculation method (Zhou et al., 2022). The percentage of inhibition of radial growth (PIRG) was calculated using the formula: *PIRG* (%) = (*C*−*T*)/*C* × 100, where *C* and *T* represent the fungal growth radii in the control and treatment groups, respectively. All strains were tested in triplicate experiments. The strain with the highest PIRG was selected for further study.

### 2.2 Identification of strain D337-11

The cultural characteristics as well as physiological and biochemical properties of strain D337-11 were analyzed according to the methods described by Zhou et al. (2022) and Bentley et al. (2002). Additionally, the morphological features of the mycelium and spores were examined using a scanning electron microscope (SEM) (Zeiss Sigma VP, Germany). The agar dilution method was employed to evaluate the resistance of strain D337-11 to 25 standard antibiotics (Chen et al., 2022). Genomic DNA was extracted using the Wizard^®^ Genomic DNA Purification Kit (Promega, Madison, WI, United States) according to the instructions. The amplified 16S *rRNA* gene sequence was aligned with those deposited in public databases and the EzBiocloud server^1^. The phylogenetic tree was reconstructed with the neighbor-joining tree algorithm using MEGA version 7.0 (Yoon et al., 2017; Kumar et al., 2016). Genomic taxonomy was further validated through comparative analyses: the OrthoANIu algorithm was employed for average nucleotide identity (ANI) calculations, and Genome-to-Genome Distance Calculator (GGDC v2.1) platform was used to determine digital DNA–DNA hybridization (dDDH) values (Meier-Kolthoff et al., 2013).

### 2.3 Fermentation and isolation of bioactive extracts

Strain D337-11 was cultured in 10 L soybean powder medium (SLM) at 28°C with shaking at 180 rpm for 9 days to obtain the fermentation solution. The mixture was leached with anhydrous ethanol in equal proportions for 2 days. After evaporation, the extracts were subjected to macroporous resin column chromatography (Diaion HP20, 100–200 μm particle size, 6 × 50 cm). The extracts were then processed through an ODS column (SiliaSphere C18, 50 μm) with successive elution of MeOH/H_2_O (5:5, 7:3, 9:1, and 10:0 v/v), yielding four fractions (Fr. HA1, Fr. HA2, Fr. HA3 and Fr. HA4). These fractions were configured into 20 g/L solutions separately. The solutions were sterilized by filtration using a 0.22-μm sterile filter. Antifungal activity was assessed using the agar dilution method, and the extract demonstrating the highest activity was selected for further investigation. The experiments were conducted in triplicate biologically. The process is illustrated in Supplementary Figure S1.

### 2.4 Evaluation of the antifungal activity of strain D337-11 extracts

Antifungal activity of the strain D337-11 extracts was determined against eight fungal pathogens obtained from various plant species: *Pestalotia mangiferae* (stored in the laboratory from mango), *Colletotrichum gloeosporioides* (ATCC 58222) from mango, *Colletotrichum acutatum* (ATCC 56815) from loquat, *Colletotrichum fragariae* (ATCC 58718) from strawberry, *Fusarium oxysporum* f. sp. *cucumerinum* (ACCC 30220) from cucumber, *Colletotrichum musae* (ATCC 96726) from banana, *Fusarium graminearum* (ATCC 46779) from wheat, and *Curvularia lunata* (ATCC 42011) from maize. A conventional spot inoculation method was used to identify the PIRG of strain D337-11, while the PIRG of the extracts from strain D337-11 was evaluated using the agar dilution method (Zhou et al., 2022). The control group consisted of untreated fungal plates.

### 2.5 EC₅₀ determination

The EC_50_ of the extracts from strain D337-11 was determined using the agar dilution method (Chen et al., 2022). Extracts were prepared in 10% (v/v) dimethyl sulfoxide (DMSO) at a concentration of 20.0 μg/mL. These extracts were then applied to potato dextrose agar (PDA) plates at various concentrations of 400, 200, 100, 50, 25, 12.50, and 6.25 μg/mL. A control was established using 10% (v/v) DMSO only. A 5-mm diameter disk of *Foc* TR4 was placed in the center of each plate and incubated at 28°C. The EC_50_ value was calculated following the method described by Van Ewijk and Hoekstra (1993). Linear regression was performed to model the relationship between virulence (Y) and the treatment dose (x). The model parameters, including intercept and slope, were estimated using ordinary least squares method. We log-transformed the concentration data and established a linear regression equation. Standard errors for the coefficients were calculated, and 95% confidence intervals for the EC_50_ values were derived using the delta method. Model adequacy was verified through residual analysis and Shapiro–Wilk normality test. Statistical significance was set at *p* < 0.05. Each treatment was conducted in triplicate.

### 2.6 Hyphal morphology and spore germination assays

A 4 mm diameter mycelial disk was obtained from the edge of a colony cultured on PDA medium containing extracts at concentrations of 50, 100, 200, 400, and 800 μg/mL. After incubation at 28°C for 24 h, hyphal profiles were observed by the light microscope. A 10% DMSO solution served as the negative control. A spore suspension of *Foc* TR4 was prepared at a concentration of 1 × 10^6^ spores/mL in a 0.05% Tween-80 solution. Subsequently, 100 μL of the spore suspension was thoroughly mixed with 100 μL of extracts at concentrations of 4 × EC_50_, 6 × EC_50_, 8 × EC_50_ and 10 × EC_50_, respectively. A 10% DMSO solution was utilized as a negative control. All experiments were performed in three replicates. Approximate 100 conidia in each field were detected.

### 2.7 Pot experiments

A pot experiment was conducted in a greenhouse maintained at 70% humidity and 28°C, to evaluate the biocontrol efficacy of strain D337-11 extracts against *Foc* TR4. Soil samples were collected from a banana orchard in Danzhou, Hainan Province (109°54′66″E, 19°44′63”N). Banana seedlings (Cavendish cultivar “Brazilian”) were transplanted into plastic pots (30 plants per replicate). The experiment consisted of three treatments: (1) Mock Control, where plants were treated with sterile water without *Foc* TR4 inoculation; (2) Disease Control, where plants were inoculated with *Foc* TR4 (1.0 × 10^5^ CFU/g soil) and treated with sterile SLM medium; (3) Biocontrol Treatment, where plants were inoculated with *Foc* TR4 (1.0 × 10^5^ CFU/g soil) and treated with D337-11 extract (2 × EC₅₀ dissolved in sterile water). *Foc* TR4 inoculation was performed according to the method described by Jing et al. (2020). Each treatment was replicated three times. Disease severity was scored using the scale described by Qi et al. (2021), with control efficacy calculated as: Control Efficacy (%) = [(Disease index of Control-Disease index of Treatment)/Disease index of Control] × 100. Stem diameter, leaf area, plant height, and fresh weight were measured at 45 days post-inoculation. Data were analyzed using one-way ANOVA followed by Tukey’s post-hoc test (SPSS v22.0, *p* < 0.05), with results expressed as mean ± standard deviation (SD).

### 2.8 Microbial community composition analysis

Rhizosphere soil samples were collected to compare microbial community structures. The E. Z. N. A.^®^ Soil DNA Kit (Omega Bio-tek) was used to extract total genomic DNA. DNA quality and quantity were assessed with a NanoDrop 2000 spectrophotometer (Thermo Fisher Scientific). The V3–V4 hypervariable regions of the bacterial 16S rRNA gene were amplified using barcoded primers 341F (5’-CCTACGGGNGGCWGCAG-3′) and 806R (5’-GGACTACHVGGGTATCTAAT-3′) and sequenced on an Illumina MiSeq PE300 platform. Raw reads were processed with the QIIME 2 pipeline (v2023.2), including quality filtering, denoising, and chimera removal. Operational taxonomic units (OTUs) were clustered using the UPARSE algorithm (version 9.2.64) at 97% sequence similarity. Alpha diversity of Chao1 richness, Shannon diversity, and Simpson’s evenness indices were evaluated. Beta diversity of Principal Coordinate Analysis (PCoA) was visualized based on Bray–Curtis dissimilarity matrices. Microbial interactions Co-occurrence networks were constructed using SparCC (Sparse Correlations for Compositional data). Kruskal–Wallis *H* test was applied to compare the significantly different genera between treatments.

### 2.9 Genome assembly and metabolite prediction of strain D337-11

The complete genome sequencing and assembly were conducted by Majorbio Bio-pharm Technology Co., Ltd. (Shanghai, China). The obtained genome sequences were quality-controlled using the fastp software. SOAPdenovov2.04 (GapCloserv1.12) was used to assemble the genome. The data generated from the Illumina platform were used for bioinformatics analysis using the I-Sanger Cloud Platform^2^. Protein-coding gene prediction employed Glimmer v3.02, while functional annotation of these genes was carried out using the NCBI Prokaryotic Genome Annotation Pipeline (Delcher et al., 2007). Biosynthetic gene clusters for secondary metabolites were identified with the online antiSMASH v4.2.0 software. The final genome sequence has been deposited in GenBank under accession number JBBMXX000000000.

### 2.10 Untargeted differential metabolomic analyses

Fermentation extracts of strain D337-11 were collected daily from day 1 to day 10 as described in Section 2.3. Antifungal activities were evaluated using the agar dilution method (see Section 2.4), and samples with the highest and lowest inhibitory activities were selected for metabolomic analysis. A blank medium control was included to account for background metabolites. For metabolite extraction, 100 μL of each extract was mixed with 400 μL of acetonitrile/methanol (1:1, v/v) containing 0.02 mg/mL internal standard (L-2-chlorophenylalanine) in 1.5 mL centrifuge tubes. Metabolomic profiling was performed on a SCIEX UPLC-Triple TOF 5600+ system at Majorbio Bio-pharm Technology Co., Ltd. (Shanghai, China) using an ACQUITY HSS T3 column (100 mm × 2.1 mm, 1.8 μm; Waters, United States). Chromatographic separation was achieved using a gradient mobile phase (0.1% formic acid in water and 0.1% formic acid in acetonitrile) at a 0.3 mL/min flow rate. Mass spectrometry was conducted in both positive and negative ion modes with a scan range of 50–1,200 m/z. Raw data were processed using SCIEX OS 1.5 and XCMS (v3.8.0) for peak alignment, retention time correction, and normalization. Metabolite identification was performed via database matching against HMDB v5.0, Metlin, and the in-house MJDB (Majorbio Database), with mass tolerance set to ±5 ppm.

Differential metabolites were identified using OPLS-DA (Orthogonal Partial Least Squares-Discriminant Analysis) in SIMCA-P v16.0. Features with a VIP > 1 and *p* < 0.05 (Student’s *t*-test) were considered significant. Functional annotation was performed using MetaboAnalyst (v5.0) for pathway enrichment analysis. Candidate metabolites were validated via the agar dilution method (Section 2.4), with inhibition percentages calculated as described in Section 2.1.

### 2.11 Isolation of naringenin from strain D337-11 extracts

To isolate bioactive metabolites, 10 liters of strain D337-11 fermentation broth obtained as described in Section 2.3 were processed. The fraction F3 with the highest antifungal activity was subjected to silica gel column chromatography using a hexane/ethyl acetate gradient from 10:1 to 1:1 in order to remove apolar impurities. The fraction (0.6 g) was further purified using Sephadex LH-20 with MeOH/H₂O gradient from 70:30 to 100% MeOH. The final purification was conducted through semi-preparative HPLC. The target compound, naringenin, was identified by matching the retention time with its UV absorbance at 288 nm (λmax). Naringenin was confirmed by ^1^H and ^13^C NMR spectroscopy.

### 2.12 Morphological and ultrastructural effects of naringenin on *Foc* TR4

A GFP-expressing *Foc* TR4 strain was used to investigate the effects of naringenin. GFP-*Foc* TR4 hyphal disk was inoculated on the PDA plate, and a sterile slide (1 cm × 1 cm) was placed 1 cm away from the mycelium edge and cultured at 28°C for 4 days. Ten microliters of sterile naringenin solution (200 μg/mL) was added on the slide and then cultured for 2 days. The morphologic changes of GFP-*Foc* TR4 mycelia were observed by fluorescence microscopy (OLYMPUS, SZX16, Japan). Furthermore, the morphological effects of naringenin were observed using a scanning electron microscope (Sigma 500/VP, Zeiss, Germany). A PDA plate of 200 μg/mL naringenin extract was attached with Foc TR4 hyphal disk and cultured at 28°C for 3 days. A sterile scalpel was used to cut a 1 cm square of mycelium edge and immersed in a centrifuge tube containing 2.5% glutaraldehyde solution. The *Foc* TR4 spore solution (1 × 10^6^ CFU/mL) and 200 μg/mL naringenin solution were mixed in equal volume and 30 μL mixed solution was placed on the glass slide. The control was equal volume of sterile water. The sample was pre-fixed in 3% fresh glutaraldehyde and post-fixed in 1% osmium tetroxide, followed by dehydration through a graded water-acetone series (50%, 70%, 80%, 90%, 95%, 100%) with each step lasting 10 min. It was then embedded in Epon 812 resin and cured at 37°C for 12 h, 45°C for 12 h, and 60°C for 24 h sequentially. Sections were cut using a Leica EM UC6 ultramicrotome, double-stained with saturated uranyl acetate and lead citrate, and observed under a Hitachi HT7700 TEM at 80 kV.

### 2.13 Prediction of target proteins of naringenin in *Foc* TR4

Molecular docking software was employed to simulate interactions between target proteins and the small molecule naringenin, predicting binding affinity based on their 3D structures. Target proteins from *Fusarium* species were identified via RCSB PDB database searches. The naringenin structure was imported into Chem3D v20.0, converted into a 3D structure, and energy-minimized using the MM2 force field. Both the ligand and core proteins were processed in AutoDock Tools v1.5.7 for hydrogen addition, charge calculation, and conversion to .pdb format. Active sites were identified with POCASA v1.1 using a 40 Å docking box. Molecular docking was performed using AutoDock Vina v1.1.2. with binding energy thresholds set at <−4.25 kcal/mol for moderate binding, <−5.0 kcal/mol for good binding, and <−7.0 kcal/mol for strong binding, to evaluate activity. To ensure the rigor of the results, we conducted both positive and negative controls: specifically, we predicted the binding of the antifungal compound phenazine to potential targets (Lu et al., 2024), and verified the binding of naringenin to common known target proteins of pathogenic fungi.

## 3 Results

### 3.1 Isolation, identification, and characterization of strain D337-11

**Figure 1:**
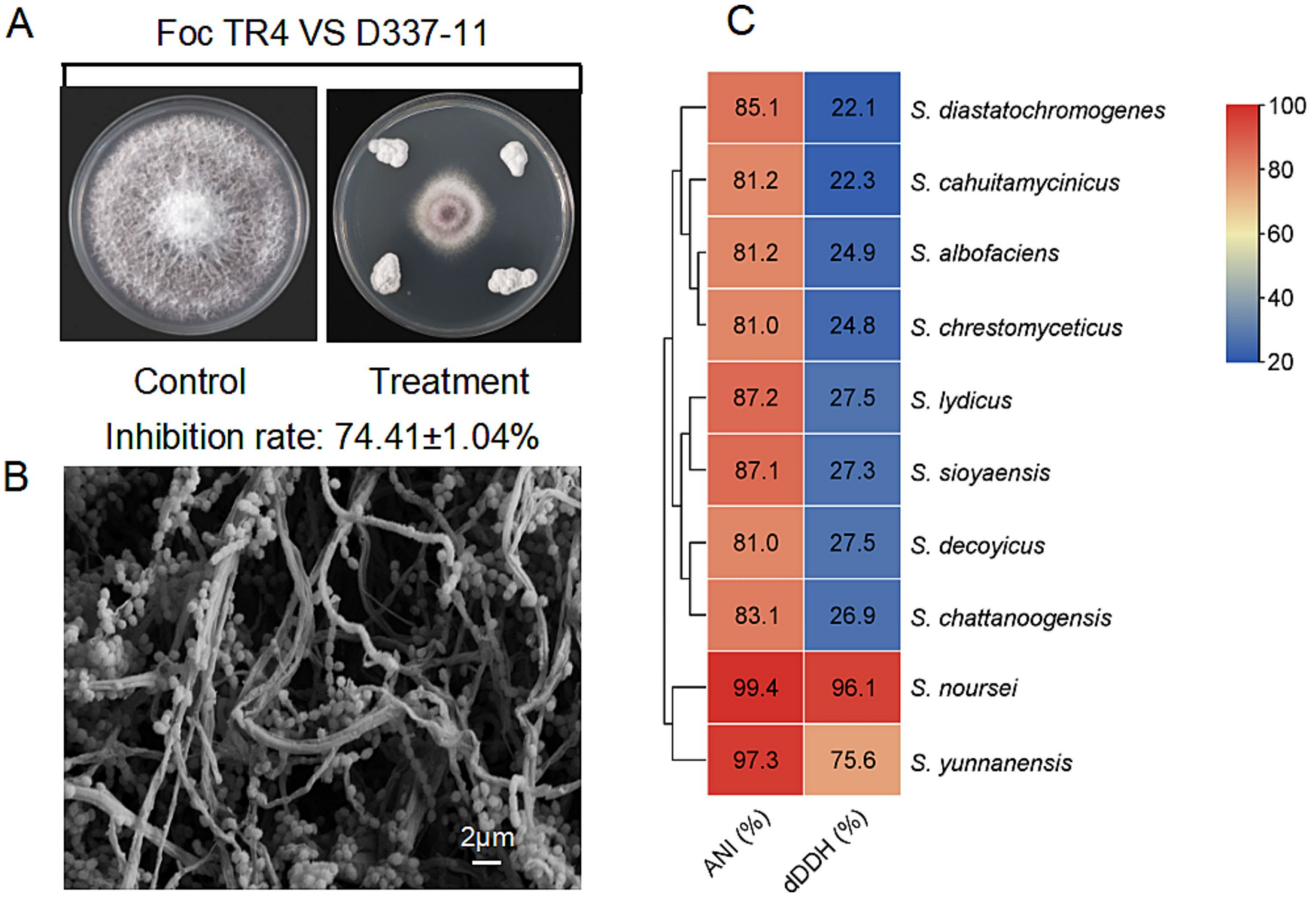
Characterization of strain D337-11. **(A)** Inhibition of mycelial growth of *Foc* TR4 by strain D337-11. **(B)** Morphological characteristics of the aerial mycelia and spores of strain D337-11 observed by SEM. **(C)** Average nucleotide identity (ANI) and Digital DNA–DNA hybridization (dDDH) comparisons between the genomes of *Streptomyces* sp. D337-11 and other closely related members of the *Streptomyces* genus.

A total of 121 actinomycete isolates were obtained from banana rhizosphere soil, with 24 strains showing significant biocontrol potential against *Foc* TR4 (see Supplementary Figure S2 and Supplementary Table S1). Among these, strain D337-11 exhibited the highest inhibitory activity, achieving 74.41% mycelial growth inhibition in plate confrontation assays and 65.75% inhibition in liquid fermentation tests (Figure 1A and Supplementary Figure S3A). Therefore, we selected this strain for further study.

Strain D337-11 demonstrated vigorous growth on ISP2, ISP5, ISP6, and ISP7 media (Supplementary Table S2), tolerated a pH range of 4.0–9.0 and up to 3% NaCl (Supplementary Table S3). Biochemical profiling revealed versatile metabolic capabilities, including phosphate solubilization, nitrogen fixation, cellulose degradation, and production of urease and esterase (Supplementary Table S3). The strain efficiently utilized a variety of carbon/nitrogen sources such as raffinose, valine, and phenylalanine (Supplementary Table S4). It displayed broad-spectrum antibiotic resistance, effectively resisting 15 tested compounds (Supplementary Table S5). SEM observations revealed filamentous hyphae and spiny spherical spores, characteristic of the *Streptomyces* genus (Figure 1B). 16S rRNA gene phylogenetic analysis placed strain D337-11 in a monophyletic clade with *S. noursei* ATCC 11455, *S. albulus*, and *S. yunnanensis* (bootstrap support >80%, Supplementary Figure S2B). When compared to *S. noursei* (ATCC 11455), the OrthoANI and dDDH values were found to be 99.44 and 96.10%, respectively, which surpassed the species delineation threshold of 95 and 70% (Figure 1C). Thus, strain D337-11 was identified as *S. noursei.*

### 3.2 Extracts of strain D337-11 exhibit antifungal activity and promote plant growth

**Figure 2:**
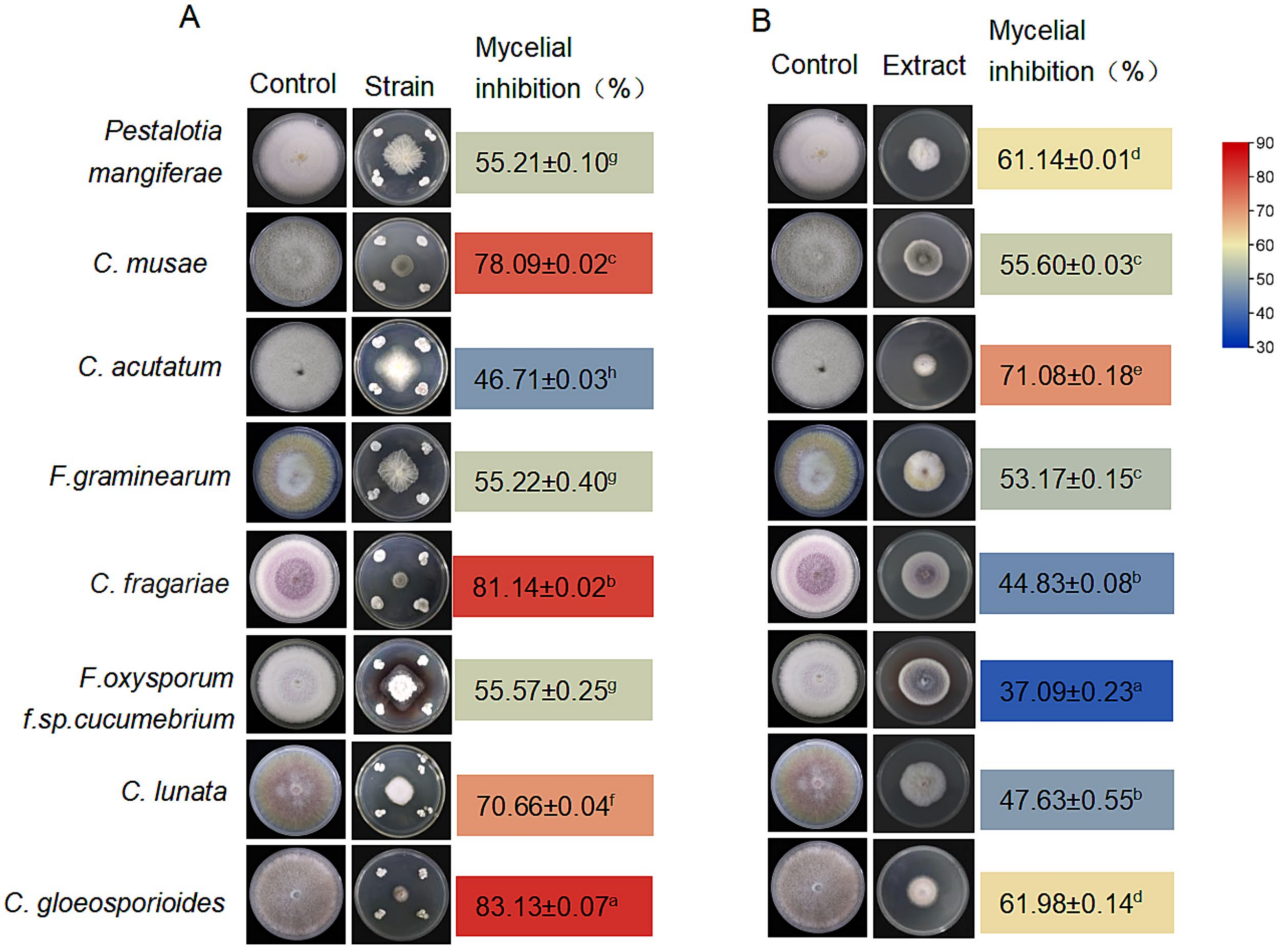
Antifungal activity of *Streptomyces noursei* D337-11. Antifungal activity of *S. noursei* sp. D337-11 **(A)** and crude extracts from *S. noursei* D337-11 **(B)** against selected phytopathogens. The letters (a–h) indicate significant differences at the *p* < 0.05 level, according to Duncan’s multiple range test, expressed as mean ± SD.

**Figure 3:**
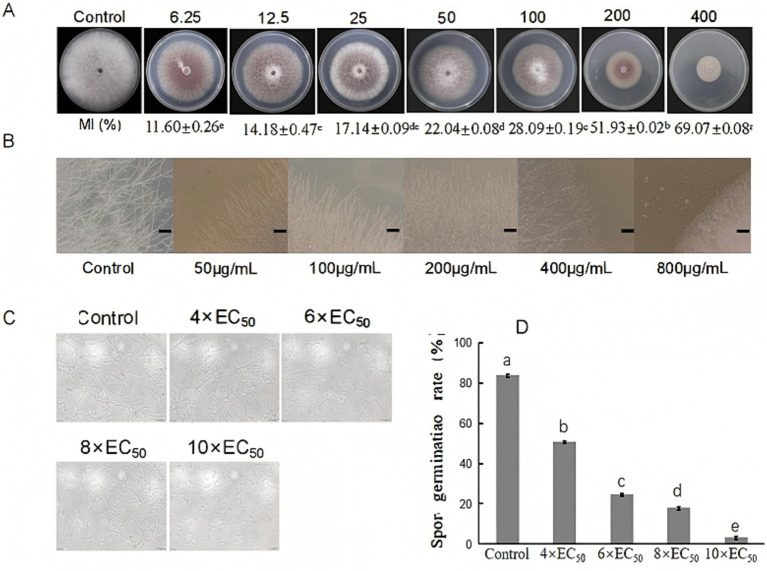
Antifungal activity of extracts from *S. noursei* D337-11 against *Foc* TR4. **(A)** Inhibition of mycelial growth at different concentrations of extracts. **(B)** Influence of extracts on hyphal morphology of Foc TR4. **(C)** Spore germination of *Foc* TR4 at different concentrations. **(D)** Spore germination rate of *Foc* TR4. Letters (a–e) represent significant differences at the *p* < 0.05 level according to Duncan’s multiple range test, expressed as mean ± SD.

The strain *S. noursei* D337-11 exhibited strong inhibitory effects against *Curvularia gloeosporioides, C. fragariae,* and *C. musae* with inhibition rates of 83.13, 81.14, and 78.09%, respectively (Figure 2A). Among the eight tested species inhibition rates ranged from 37.09 to 71.08% (Figure 2B), confirming its broad-spectrum activity. To further assess the activity of extracts against *Foc*, various concentrations of methanol were used to prepare crude extracts. The fraction obtained with 90% methanol (Fr. HA3) showed the highest activity, achieving a 64.71% inhibition rate (Supplementary Figure S3C). This fraction was then selected for further studies. As shown in Figure 3A, the inhibitory effects against *Foc* TR4 increased in a dose-dependent manner. The linear regression model for virulence was calculated as follows: *Y* = 2.8755 + 0.9124x, with a coefficient of determination (*R*^2^) of 0.9480 (*p* < 0.01), indicating that 94.8% of the variability in virulence was explained by the independent variable. The EC_50_ value was determined to be 213.11 μg/mL (95% confidence interval: 205.43–220.79 μg/mL). All statistical analyses were performed using R software (version 4.2.1), and confidence intervals were calculated using the delta method.

The mycelia of *Foc* TR4 treated with *S. noursei* D337-11 extracts became denser as the concentration increased, while the control mycelia remained intact and developed normally. At a concentration of 50 μg/mL, the mycelia appeared to break apart; they became inseparable at 200 μg/mL and tended to melt at 400 μg/mL of crude extracts. At 800 μg/mL, the mycelia completely dissipated and failed to grow outwards (Figure 3B). The spore germination rate decreased gradually with increasing concentration of the extract. At 4 × EC_50_, the germination rate was 50.52%; it dropped to 24.17% at 6 × EC_50_; and further declined to 18.22% at 6 × EC_50_. At 10 × EC_50_, the germination rate was only 3.06%, whereas the control exhibited a germination rate of 84.25%. This indicates that the crude extract of strain D337-11 significantly inhibited the germination of *Foc* TR4 (Figures 3C,D).

**Figure 4:**
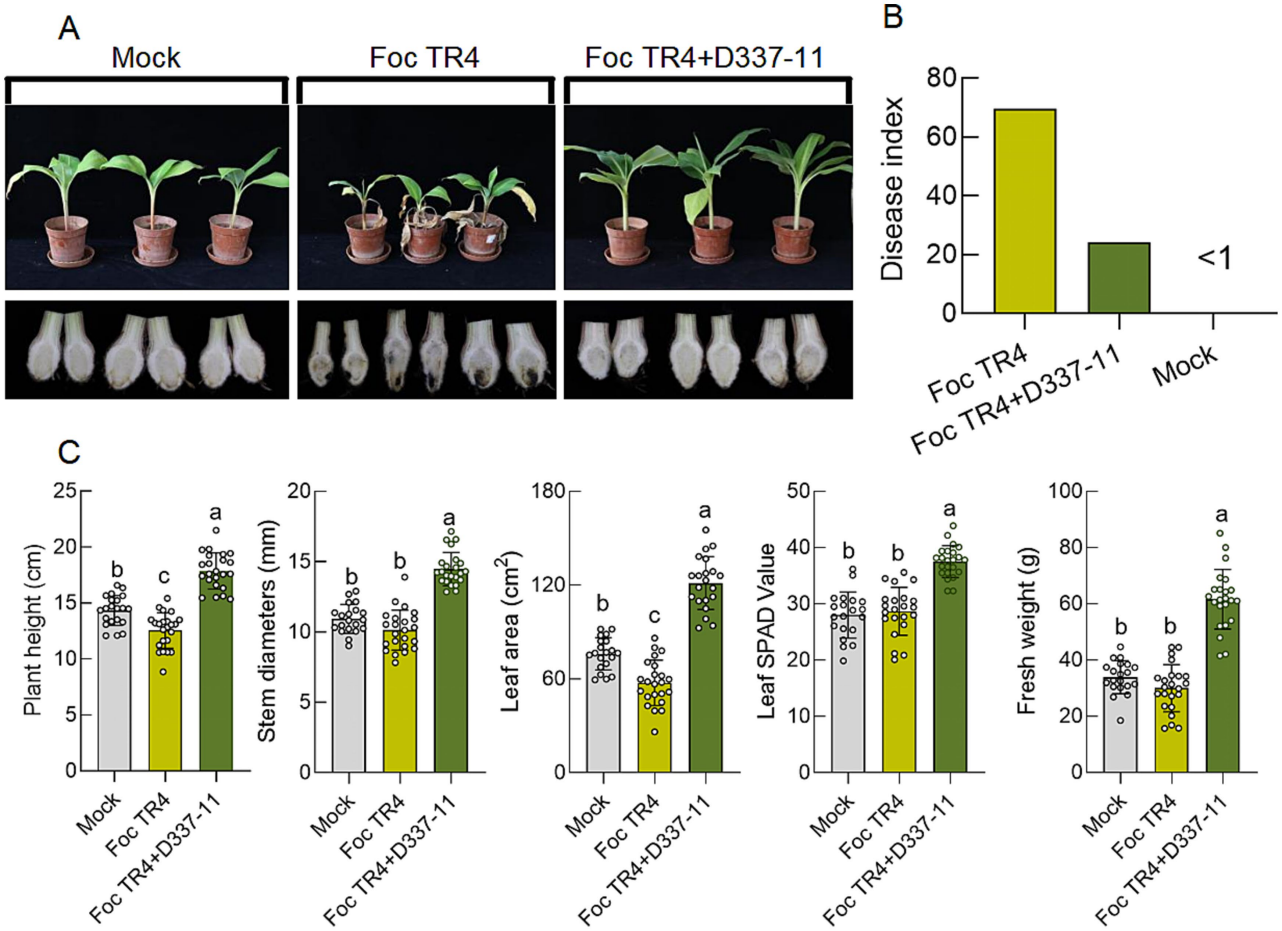
Effects of *S. noursei* D337-11 extracts on disease control and banana growth promotion. **(A)** Chlorotic symptoms of banana seedlings (upper panels) and dissection of the corresponding corms (lower panels). **(B)** Disease control efficiency (65.34%), represented through statistical analysis of the disease index of banana seedlings. **(C)** Growth promotion assessment and different letters represent significant differences at the *p* < 0.05 level.

In planta trials demonstrated that treatment with D337-11 extract reduced *Fusarium* wilt disease index from 69.70 ± 3.21 (*Foc* TR4 control) to 24.16 ± 2.85, corresponding to 65.34% biocontrol efficacy compared to the control group (Figures 4A,B). Importantly, the treatment also enhanced systemic growth, leading to a 42.80% increase in stem diameter, a 111.06% increase in leaf area, a 41.07% increase in plant height, a 27.74% increase in chlorophyll content, and substantial increases of 105.16% in fresh weight (Figure 4C). These findings strongly suggest that strain D337-11 also possesses growth-promoting properties.

### 3.3 Extracts of strain D337-11 alter rhizosphere microbiome structure

**Figure 5:**
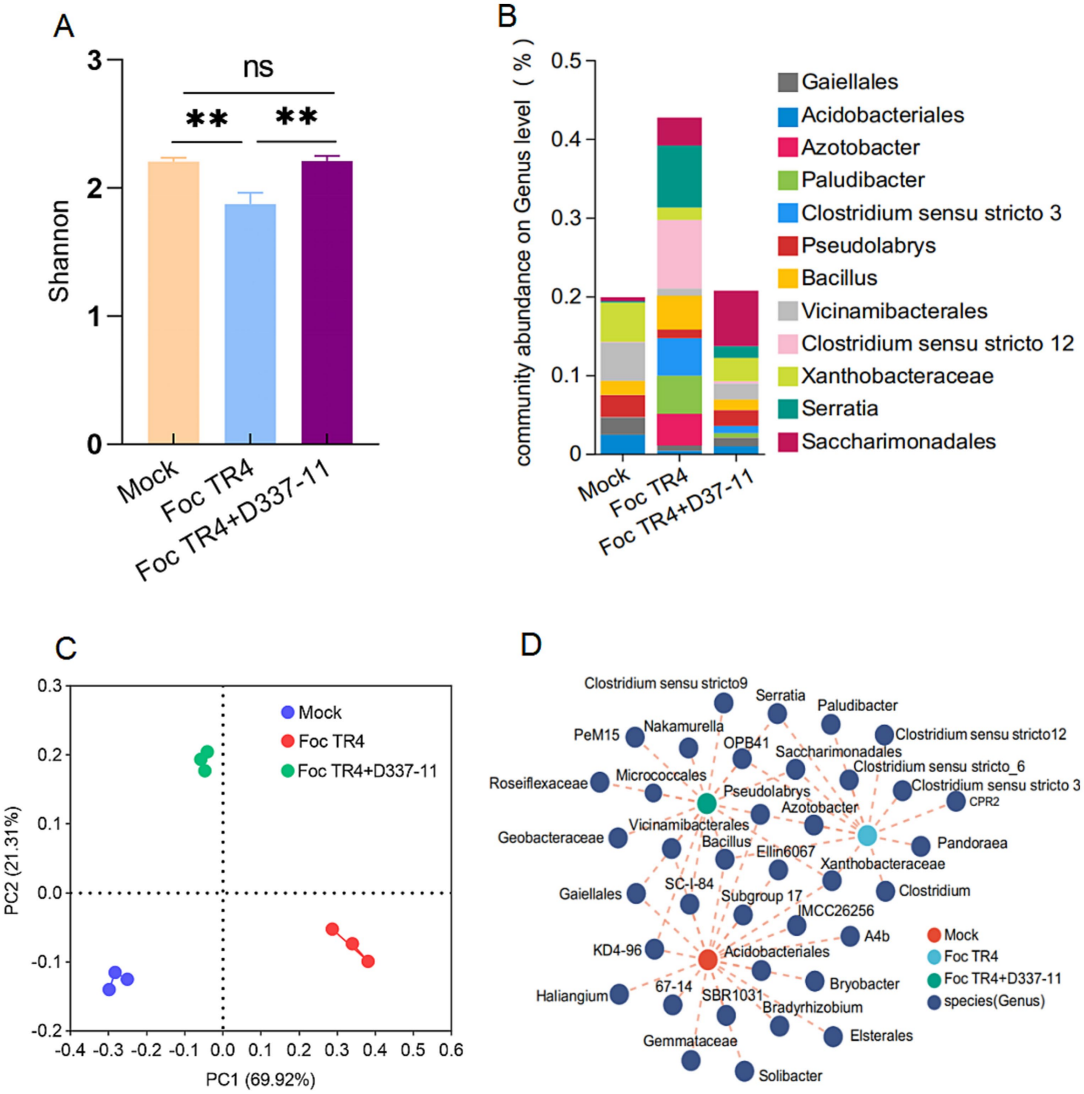
Effects of *S. noursei* D337-11 extracts on the soil bacterial community. **(A)** Simpson index of bacterial community based on ANOSIM tests and **denotes *P* < 0.001. **(B)** Principal coordinate analysis (PCoA) of bacterial community composition across all rhizosphere soil samples. **(C)** Relative abundance of bacterial genus in the rhizosphere soil. **(D)** Co-occurrence network analysis of bacterial taxa at the genus level.

The bacterial community in the soil treated with *S. noursei* D337-11 extracts showed an increasing trend in bacterial abundance compared to the *Foc* TR4 group (Figure 5A). Principal Coordinate Analysis (PCoA) indicated a clear separation of bacterial community composition, with 69.92% variance explained in the first component (PC1) and 21.30% in the second component (PC2) (Figure 5C). The main genera identified based on the relative abundance included *Gaiellales, Acidobacteriales, Azotobacter, Paludibacter, Clostridium sensu stricto 3, Pseudolabrys, Bacillus, Vicinamibacterales, Clostridium sensu stricto 12, Xanthobacteraceae, Serratia,* and *Saccharimonadales* (Figure 5B). Notably, *Saccharimonadales,* a taxon involved in carbon cycling and pathogen antagonism, was significantly enriched in the *S. noursei* D337-11 treatment (Supplementary Figure S4). Co-occurrence network analysis revealed that the bacterial network nodes were predominantly composed of the genus *Xanthobacteraceae, Serratia, Clostridium Pseudolabrys, Bacillus, and Saccharimonadales* (Figure 5D). Among the three treatments (see Materials and Methods, section 2.7), the node degree and Degree_Centrality in the Mock (963, 0.83) and *S. noursei* D337-11 treatments (945, 0.82) was higher than in the *Foc* TR4 group (926, 0.80), indicating that the disease affected the stability of the network composition. However, the *S. noursei* D337-11 treatment played an important role in network stability.

### 3.4 Identification and structure elucidation of naringenin, a key antifungal compound from the strain D337-11

After quality control 1,391,474,734 bp of clean reads were obtained. The coverage (%) based on reads mapping was 97.56%. After assembling, the total scaffolds of *S. noursei* D337-11 were 120 and the total based scaffold was 9,909,300 bp, with N50 and N90 values of 228,599 and 66,466, accounting for 2.31 and 0.67% of the genome (Supplementary Figure S5A). The G + C content of the genome is 71.71%. Among the functional annotations in the Cluster of Orthologous Groups (COG), the largest number of annotated genes is related to metabolism, with 263 genes involved in secondary metabolite synthesis, transport, and catabolic metabolism, accounting for 4.14% (Supplementary Figure S5B), exceeding the average of *Streptomyces* genomes (2.8 ± 0.6%, *p* = 0.017, one-sample *t*-test) (Aigle et al., 2014). KEGG analysis revealed that genes for metabolic pathways are among the most abundant, totaling up to 3,084 (Supplementary Figure S5C). A total of 58 biosynthetic gene clusters responsible for secondary metabolites were identified in the genome of strain D337-11. These include 18 type I polyketide synthase (PKS) gene clusters, 7 non-ribosomal peptide synthetase (NRPS) gene clusters, 3 NRPS-like gene clusters, and 2 siderophore gene clusters (Supplementary Table S6). Using the antiSMASH software in conjunction with GenBank for alignment, we observed over 90% similarity in the gene clusters associated with producing six chemical compounds (Supplementary Figure S6). Notably, the phylogenomic analysis revealed 100% synteny with naringenin biosynthetic gene clusters (BGCs) in *Streptomyces clavuligerus*, strongly supporting functional conservation.

**Figure 6:**
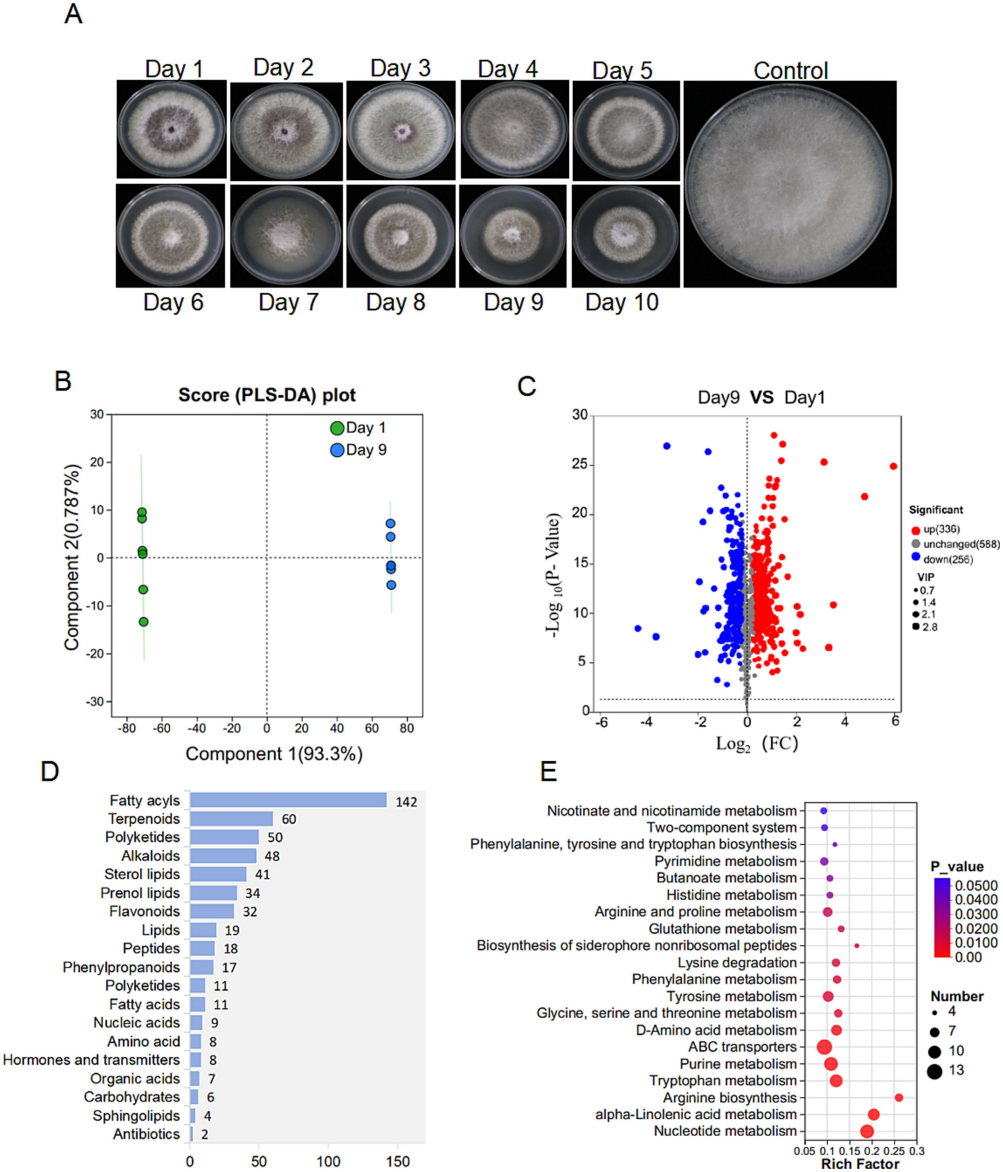
Metabolome analysis of strain D337-11 metabolites. **(A)** The metabolic activity of *S. noursei* D337-11 during different days of culture. **(B)** PLS-DA analysis of different groups. **(C)** Volcanic map of differential metabolites. Each dot represents a metabolite. The abscissa represents the group contrast ratio of each material change (using a base-2 logarithm), while the vertical ordinate indicates the *p*-value from the t-test (on a log base-10 scale). Red dots represent up-regulated metabolites, blue dots represent down-regulated metabolites, and gray dots indicate no significant difference. **(D)** Classification of overall metabolites according to KEGG compounds. **(E)** KEGG enrichment analysis of differentially expressed metabolites.

**Figure 7:**
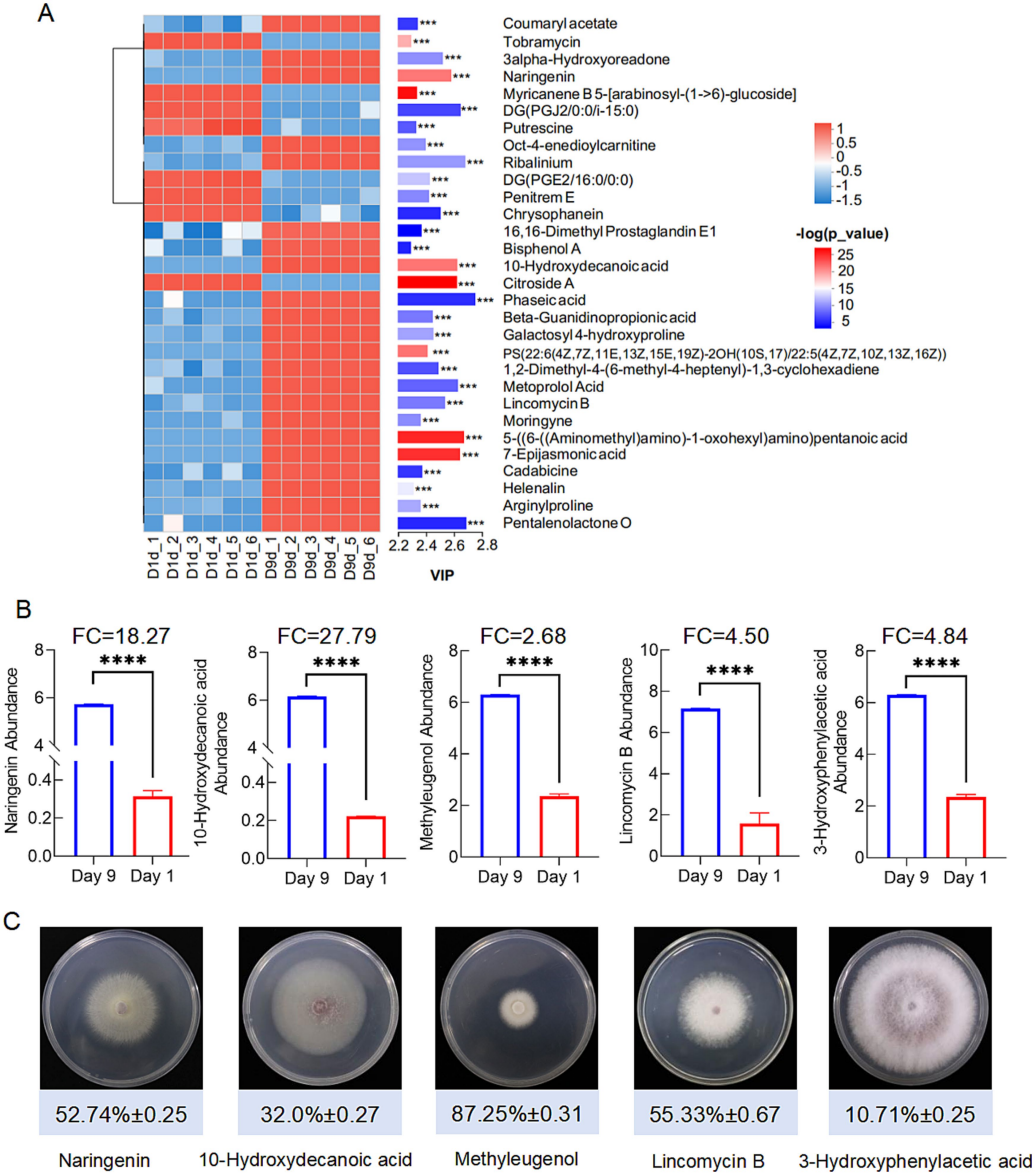
Screening of significant difference metabolites. **(A)** Significant up-regulated and down-regulated metabolites according to Vip-value. **(B)** Screening antifungal compounds according to FC and Vip-value. ****denotes *P* < 0.0001. **(C)** Antifungal activities of 5 compounds identified from the metabolites.

The extracts from the 9th day and the first day exhibited significant differences in inhibitory activity with 63.28 and 6.55%, respectively (Figure 6A). Principal component analysis (PLA-DA) confirmed the significant differences in metabolic profiles at these two fermentation time points (Figure 6B). Volcano plot analysis identified 336 up-regulated and 256 down-regulated metabolites in the 9th day extract (Figure 6C). KEGG annotation classified 527 metabolites into several major categories including fatty acyls (142), terpenoids (60), polyketides (50), and alkaloids (48) (Figure 6D). Furthermore, enrichment analysis linked the differential metabolites to critical pathways, including amino acid metabolism, secondary metabolite biosynthesis, and carbohydrate/energy metabolism (Figure 6E). After multiple testing, we identified 100 significantly up-regulated metabolites under the metabolomics criteria of FC ≥ 2, *p* < 0.05, VIP ≥ 1. Coumaryl acetate, 3-alpha-hydroxyoreadone, and naringenin were considered as the top candidates enriched in day 9 extracts by VIP-ranked heatmap (Figure 7A). Following, we selected the top 35 fold change (FC)-ranked metabolites for antifungal validation (Supplementary Table S7). After validation, five substances including naringenin, 10-hydroxydecanoic acid, lincomycin B, methyleugenol and 3-hydroxyphenylacetic acid showed anti-*Foc* TR4 activities. The inhibition rates were 52.74, 32.00 55.33, 87.25, and 10.71%, respectively (Figure 7C). Notably, naringenin and 10-hydroxydecanoic acid exhibited substantial fold changes (FC = 18.27 and 27.79, respectively) (Figure 7B).

**Figure 8:**
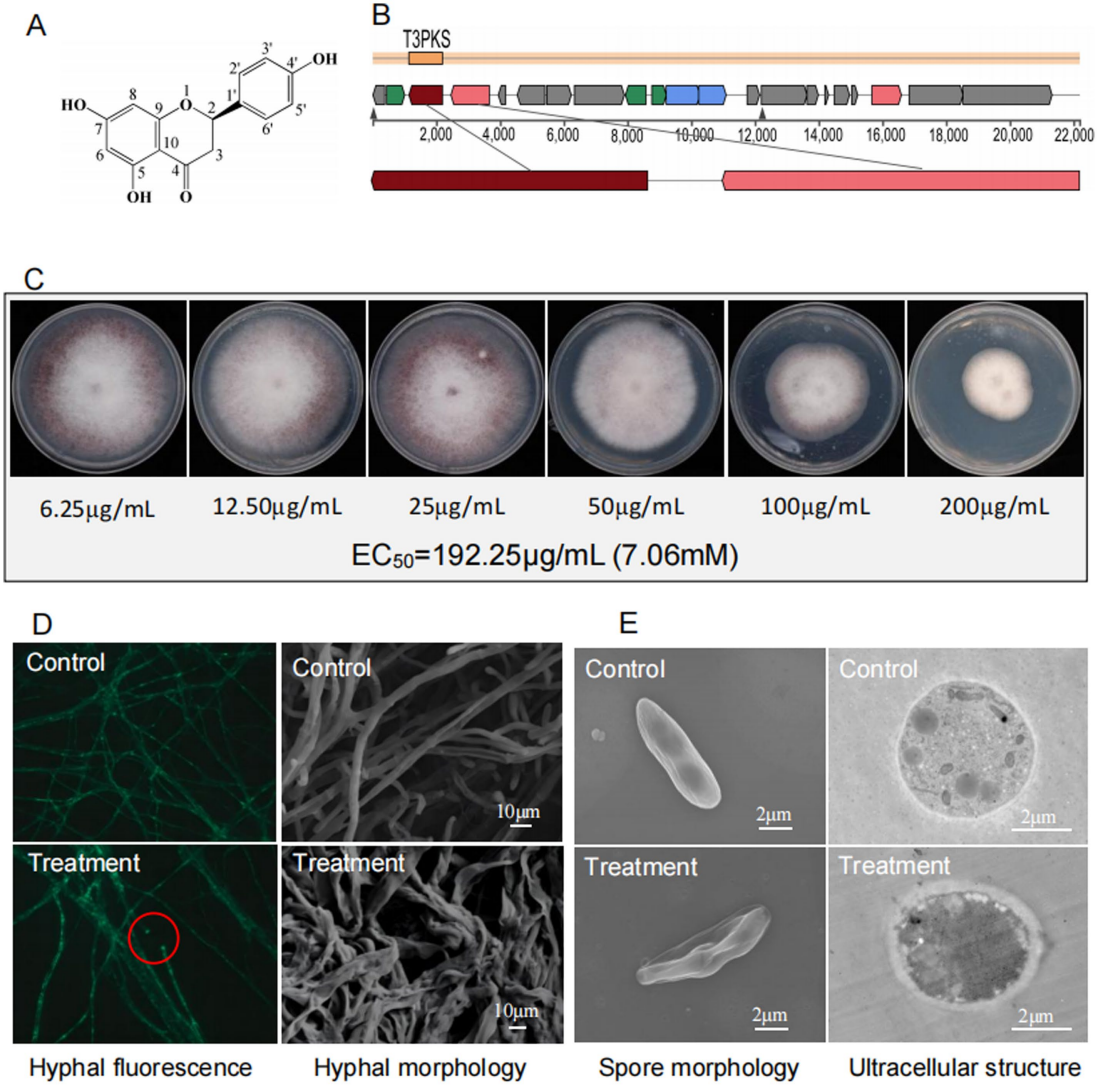
**(A)** Chemical structure of naringenin. **(B)** Genomic region of the gene cluster for the compound naringenin in *S. noursei* D337-11 and compared to MIBiG database using region-to-region comparison in antiSMASH. **(C)** Determination of EC_50_ for naringenin against *Foc* TR4. **(D)** Morphological characteristics of *Foc* TR4 hypha treated with naringenin under fluorescence microscope (40× objective lens) and SEM, respectively. **(E)** Morphological profiles of spores and cellar ultrastructure of *Foc* TR4 treated with naringenin revealed by TEM.

To validate naringenin as a bioactive component, we purified the compound from extracts of strain D337-11 using repeated preparative HPLC. From this process, we isolated a high-purity fraction with purity reaching 97% (retention time: 5.676 min) (Supplementary Figure S11) and yielded 28.42 mg of crystalline naringenin after lyophilization. Structural characterization via ^1^H-/^13^C-NMR (Supplementary Figures S7, S8) identified 15 distinct carbon signals consistent with the flavonoid backbone of naringenin. HR-ESI-TOF MS analysis further confirmed the molecular formula (C_15_H_12_O_5_, [M + H]+ ion at m/z 273.10; calculated 272.85) (Figure 8A and Supplementary Table S8). In addition, AntiSMASH analysis revealed 100% homology between the naringenin-associated gene cluster in our strain and that of *Streptomyces clavuligerus* (Figure 8B), strongly suggesting that naringenin is a primary antimicrobial compound and the Pfam of naringenin in *Streptomyces* sp. D337-11 was illustrated in Supplementary Figure S9. These findings underscore the need for further functional validation of this bioactive metabolite.

### 3.5 The antifungal mechanisms of naringenin involve disrupting the membrane integrity and targeting trypsin in *Foc* TR4

Naringenin exhibited dose-dependent antifungal activity against *Foc* TR4 with an EC_50_ of 192.25 μg/mL (7.06 mM) (Figure 8C). Microscopic observation revealed severe hyphal damage, including fusion points, irregular diameters, and surface collapse (Figure 8D). TEM observations revealed profound cellular disruption including spores displaying extensive wrinkling and cytoplasmic leakage, hyphal cell walls exhibiting thickened (250 nm vs. 35 nm in controls), degradation of organelles, and disorganized cytoplasmic matrices (Figure 8E).

**Figure 9:**
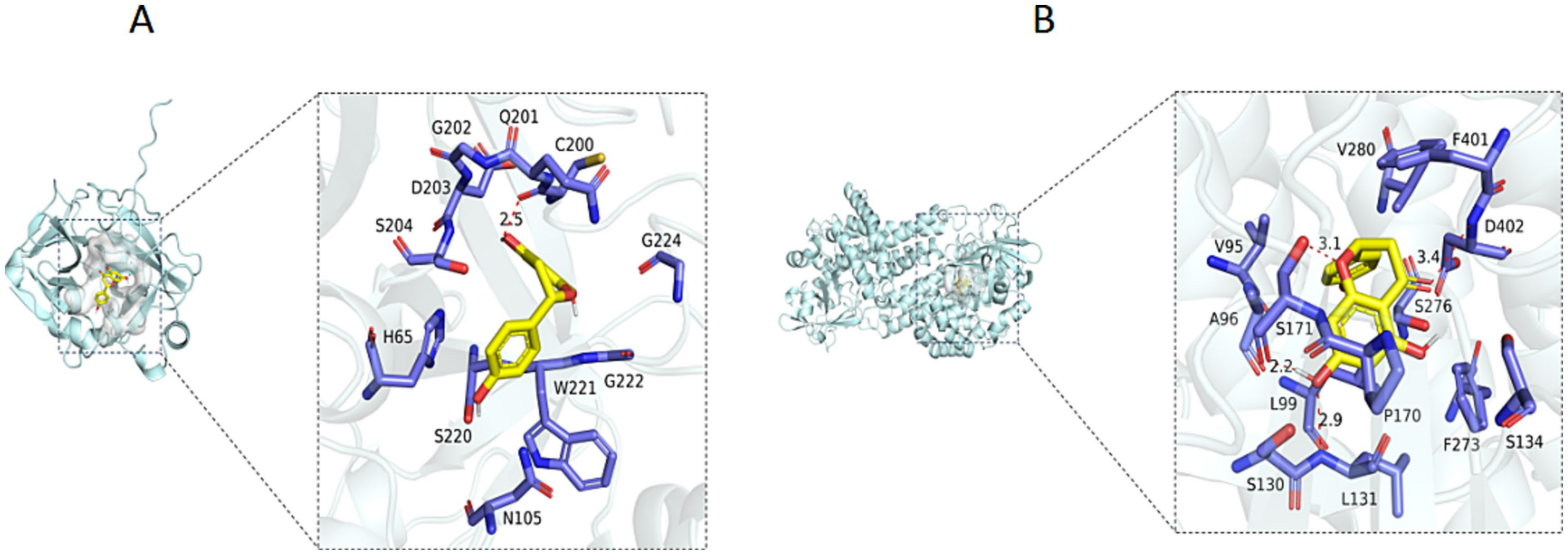
**(A)** 3D diagram of binding of Naringenin to the Trypsin Protein (NIRZA7). **(B)** 3D diagram of binding of Naringenin to the Nitroalkane oxidase Protein (N1R9V2).

Furthermore, naringenin showed the strongest binding affinity to trypsin (TRYP_FUSOX) with a binding energy of −9.9 kcal/mol and moderate interaction with nitroalkane oxidase (NAO_FUSOX) at −8.7 kcal/mol (Supplementary Figure S10). Homologs in *Foc* TR4 were identified via UniProt (P35049 and Q8X1D8): Trypsin homolog N1RZA7 and nitroalkane oxidase homolog N1R9V2. Docking with these homologs yielded the binding energies of −6.6 kcal/mol for trypsin N1RZA7 and −8.4 kcal/mol for nitroalkane oxidase N1R9V2. Key interacting residues for trypsin N1RZA7 and nitroalkane oxidase N1R9V2 were shown in Figures 9A,B, respectively. The binding energy between the small molecule ligand Phenazine and the protein Trypsin (TRYP_FUSOX, P35049) was determined to be −6.4 kcal/mol. Furthermore, the optimal binding energy between the small molecule ligand naringenin and the protein Succinate Dehydrogenase (W9I226_FUSOX, W9I226) was −6.5 kcal/mol (Chen et al., 2024b). However, the binding energy between “Phenazine and the protein Trypsin” as well as “Naringenin and the protein Succinate Dehydrogenase” were relatively low, and there were no hydrogen bond interactions between the protein and the small molecule, resulting in relatively weak inhibitory activity. These findings demonstrated that the small molecule ligand naringenin exhibited substantial binding capacity with both trypsin and nitroalkane oxidase, with the highest binding affinity observed for nitroalkane oxidase.

## 4 Discussion

Bananas are a significant fruit and staple crop in tropical and subtropical regions. Nevertheless, the industry’s progress has been significantly impeded by the occurrence and spread of *Fusarium* wilt in bananas. Biocontrol strategies that utilize antagonistic microbes may offer eco-friendly solutions to this problem. Previous research has identified several microbial taxa, including *Pseudomonas, Streptomyces,* and *Flavobacterium,* that are associated with the suppression of plant diseases (Chen et al., 2024a; Zhou et al., 2022; Gu et al., 2020). Biocontrol microorganisms can not only produce metabolites with antibacterial activity, but also occupy competitive niche advantage through synergistic interaction with microorganisms in soil to affect microbial community structure (Gu et al., 2020). Our previous research indicated that *Streptomyces* and its naturally antibiotics played a crucial role in establishing a stable, disease-suppressive soil microbial community, thereby decreasing the incidence of banana *Fusarium* wilt. However, the identification of novel bioactive metabolites remains a challenge. In our present study a systematic approach that combines ecological sampling, multi-omics analysis, and functional validation was proposed to solve this problem.

The enrichment of *Streptomyces* in disease-suppressive soils has been well documented, but the underlying mechanism is not clear (Juraschek et al., 2022). Our previous research showed that *Streptomyces* and their naturally produced antibiotics play a crucial role in forming a stable, disease-suppressive soil microbial community, thus reducing the incidence of banana *Fusarium* wilt (Zhou et al., 2019). In our present research, we isolated 121 strains of actinomycetes from banana rhizosphere soil, including 24 antagonist strains. The strain of D337-11 exhibited broad-spectrum activities. This supports the hypothesis that *Streptomyces* spp. act as “keystone inhibitors” in pathogen-restrictive microbiomes. Notably, pot experiments showed that strain D337-11’s extract exhibited dual function to suppress the fungal pathogen *Foc* TR4 with efficacy of 65.35% and plant growth promotion efficiency of 32%. This evidence proves the emerging concept of *Streptomyces*-mediated rhizosphere immunity, characterized by a combination of direct antagonism and indirect host priming. By integrating phenotypic, physiological, and biochemical characteristics with whole-genome information, we identified strain D337-11 as *S. noursei*. Although previous studies have indicated that this species can produce antibiotics against fungal pathogens (Gong et al., 2006; Liu et al., 2024; Bankar and Singhal, 2010), the specific mechanisms of action against *Foc* TR4 have not yet been reported.

To further investigate the antifungal effects and mechanisms of *S. noursei* strain D337-11, we obtained fermentation extracts through large-scale fermentation and subsequent column chromatography (Fan et al., 2023). The results indicated that these extracts significantly inhibited hyphal growth and spore germination at concentrations exceeding 200 μg/mL. Similar effects were observed in the hyphae of *F. oxysporum* after treatment with extracts from *S. bikiniensis* HD-087 (Gang et al., 2024) and *S. yongxingensis* sp. nov JCM 34965 (Chen et al., 2022). Analysis of the soil microbial community revealed that the strain extracts stabilized the microbial ecological structure disrupted by *Foc* TR4. Importantly, its ability to restore *Saccharimonadales* populations in pathogen-infested soils may suggest a novel ecological strategy: resuscitating native microbial sentinels to outcompete invaders. So, our next research will focus on revealing the mechanisms at the microbial ecological level.

The chemical complexity of metabolomes in *Streptomyces* has long hindered targeted discovery (Ayswaria et al., 2020). In this study, we employed a genome-guided differential metabolomics strategy to address this challenge. Through the combined analysis of metabolomics and genomics, we found that naringenin was enriched in day 9 extracts with FC value reached 18.27. Furthermore, In addition, AntiSMASH analysis revealed 100% homology of naringenin-associated gene cluster in this strain. So, we predicted that naringenin was the main antifungal substance. To prove our prediction, we used a bioactive substance-oriented separation method to isolate and identify a key antifungal compound of naringenin. This compound is a plant flavonoid that has not been previously reported as an antifungal agent in microbes (Atoki et al., 2024; Motallebi et al., 2022). The available evidence demonstrates that naringenin, as an herbal medicine, possesses significant pharmacological properties, such as anti-inflammatory, antioxidant, neuroprotective, hepatoprotective, and anti-cancer activities (Motallebi et al., 2022). However, there are no reports documenting its effectiveness as an antifungal agent, particularly against soil-borne pathogenic fungi. Moreover, this study provides the first report of antifungal naringenin produced by *Streptomyces.* Besides naringenin, we also identified four anti-*Foc* TR4 compounds of 10-hydroxydecanoic acid, lincomycin B, methyleugenol and 3-hydroxyphenylacetic acid by differential metabolome. These results suggested that the antifungal effect of the strain may be the result of a combination of active components, but the main disease resistance component is naringenin.

In this study, we used the combined cross-analysis method of metabolomics and genomics to obtain active metabolites. Firstly, the differential metabolome was applied to obtain the metabolites significantly upregulated at two fermentation time points of the strain (accumulation of naringenin, FC = 18.27), so as to narrow down the range of compound screening. Then, the target metabolite was locked by gene cluster analysis, which greatly improved the separation efficiency of antifungal substances (Álvarez-Álvarez et al., 2015). Meanwhile, our study identified that naringenin targets trypsin (N1RZA7) and nitroalkane oxidase (N1R9V2) in *Foc* TR4 through molecular docking. This suggests that naringenin exerts antifungal effects by inhibiting key enzymatic activities (Batth et al., 2024). Trypsin is likely involved in proteolytic processes critical for fungal infection, while nitroalkane oxidase may modulate toxin metabolism. The dual inhibition of these enzymes could disrupt essential fungal physiological functions. Therefore, we will further investigate the mechanism of target enzymes, optimize the structure of compound and elucidate the ecological mechanism of disease-suppressive soil for developing new antifungal methods to control *Foc* TR4. Furthermore, our pot experiment showed the growth promotion effect of strain D337-11 in, the stem diameter, leaf area, plant height, chlorophyll content and fresh weight. We speculate that the major mechanism was the inducible components in the strain extract. Previous studies indicated that jasmonates (JAs), which were key phytohormones regulating plant growth and stress responses (Saito et al., 2023; Ruan et al., 2019; Tang et al., 2020). The metabolomic results showed that 7-epijasmonic acid was significantly enriched in *S. noursei* D337-11 cultures as the top differential metabolite, the FC value reached 62.74. So, we believe that jasmonic acid was responsible for the growth promotion effect. However, further in-depth research is needed to elucidate the promotion mechanisms of *S. noursei* D337-11.

## 5 Conclusion

In conclusion, this study provides the first evidence of *Streptomyces*-produced flavonoids with antifungal functionality, underscoring disease-suppressive microbiomes as potential reservoirs for novel agro-antibiotics. The combined application of metabolomics, genomics and functional validation improved the efficiency of compound separation and identification, and we also established a paradigm for the discovery of new antifungal agents in plant-microbial ecosystems.

## Data Availability

The datasets presented in this study can be found in online repositories. The names of the repository/repositories and accession number(s) can be found: https://www.ncbi.nlm.nih.gov/genbank/, JBBMXX000000000.

## Footnotes

^1^ https://www.ezbiocloud.net/identify

^2^ www.i-sanger.com
