# Supplementary material for: Biocontrol potential of newly isolated Streptomyces noursei D337-11 from disease suppressive soil and its metabolites against Fusarium oxysporum f. sp. cubense in banana plants
Source: Front Microbiol. 2025 Aug 26;16:1655103. doi: 10.3389/fmicb.2025.1655103 (PMC12417419; doi:10.3389/fmicb.2025.1655103)
Supplement: Supplementary file 1 [file Table_1.docx]

**Supplementary Information**

# **Biocontrol Potential of Newly Isolated *Streptomyces noursei* D337-11 from Disease Suppressive Soil and Its Metabolites Against *Fusarium oxysporum* f. sp. *cubense* in Banana Plants**

**Dengbo Zhou^1†^, Xinxin He^1†^, Yufeng Chen^1^, Chunting Li^1^, Wei Wang^1^, Zhiqiang Pan^2^, Yankun Zhao^1^, Yongzan Wei^1^, Junting Feng^1^, Miaoyi Zhang^1^, Dengfeng Qi^1^, Xiaojuan Li^1^, Kai Li^1^,Tao Jing^1*^, Jianghui Xie^1*^**

# ^1^National Key Laboratory for Tropical Crop Breeding,S anya Research Institute and Institute of Tropical Bioscience and Biotechnology, Chinese Academy of Tropical Agricultural Sciences, Sanya,572025, China

^2^Agricultural Research Service, Natural Products Utilization Research Unit, U.S. Department of Agriculture, University of Mississippi, University, Mississippi 38677, United States

^†^These authors share first authorship

**^*^ Corresponding authors**

Dr. Tao Jing

Email: sjs1227@163.com

Dr. Jianghui Xie

Email: [xiejianghui@itbb.org.cn](mailto:xiejianghui@itbb.org.cn)


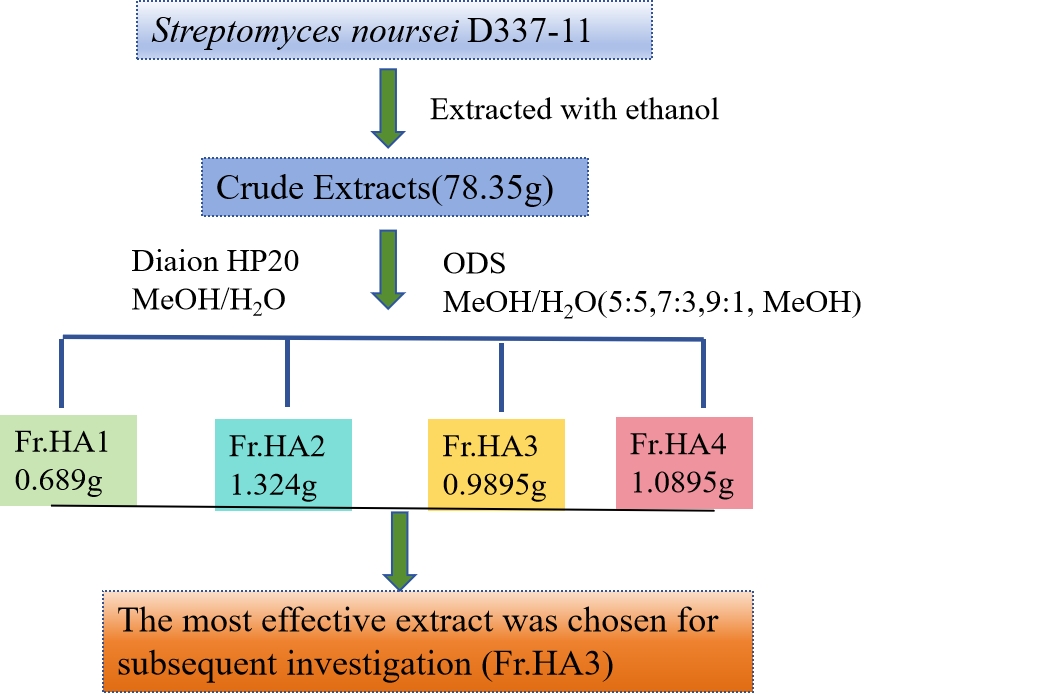


**Figure S1.** Separation process of *Streptomyces noursei* D337-11 extracts.


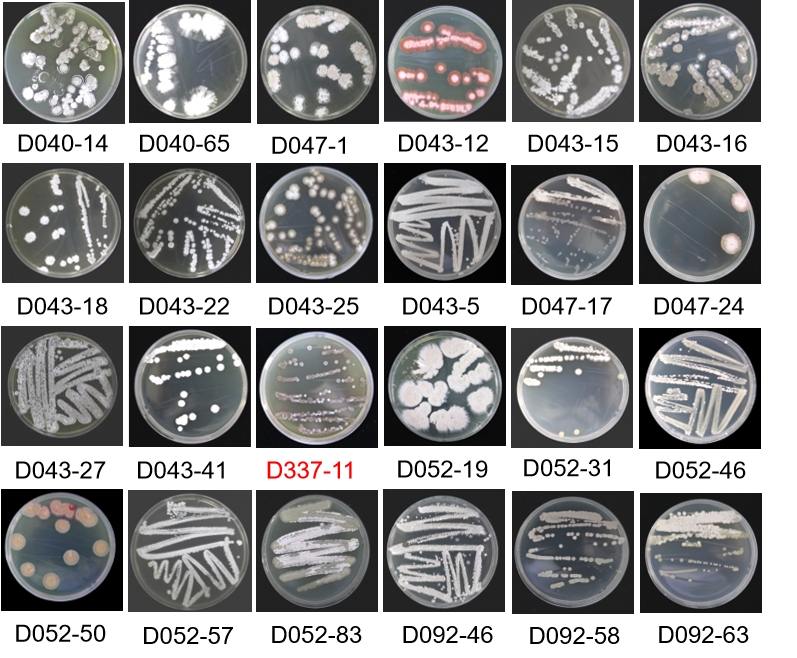


**Figure S2**. Cultivation characteristics of antagonistic strains

B

A


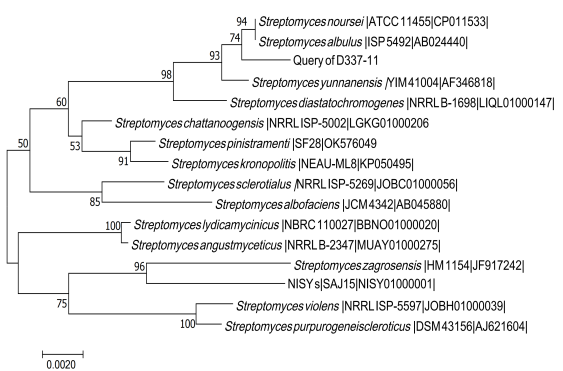


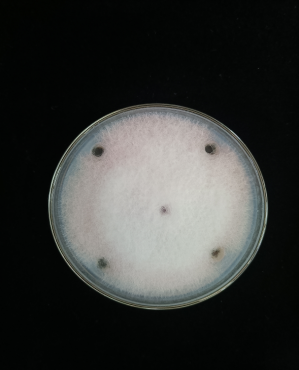

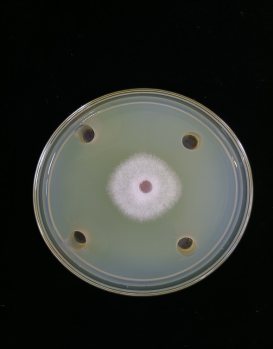


*Foc* TR4 VS D337-11 extract

Control

Inhibition rate: 65.75±0.29%

Treatment

C

Control

16.17±0.01^d^

21.53±0.10^c^

64.71±0.08^b^

42.71±0.15^a^


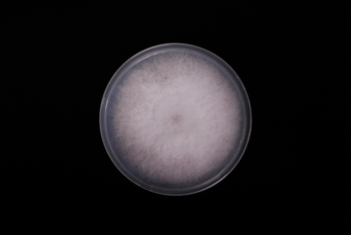

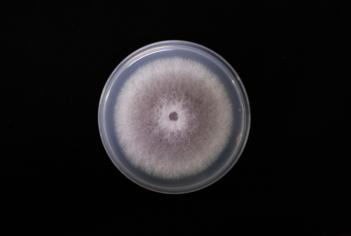

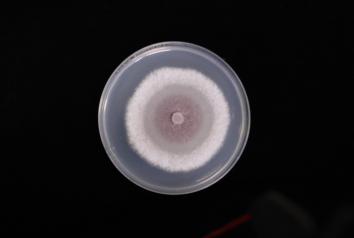

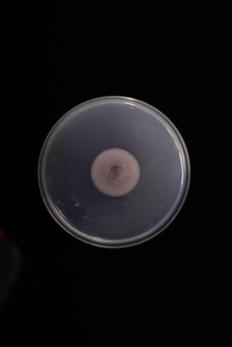

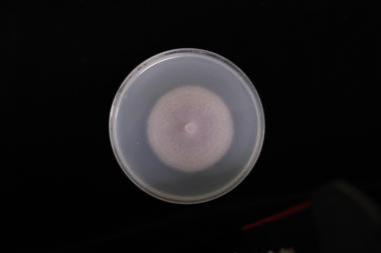


50%

70%

90%

100%

MI (%)

**Figure S3.** (A) Strain D337-11 extracts inhibiting mycelial growth of *Foc* TR4. (B) Phylogenetic tree of strain D337-11 based on 16S rRNA gene sequence analysis. (C) Antifungal effects of different methanol fractions of crude extracts from *Streptomyces* sp. D337-11.


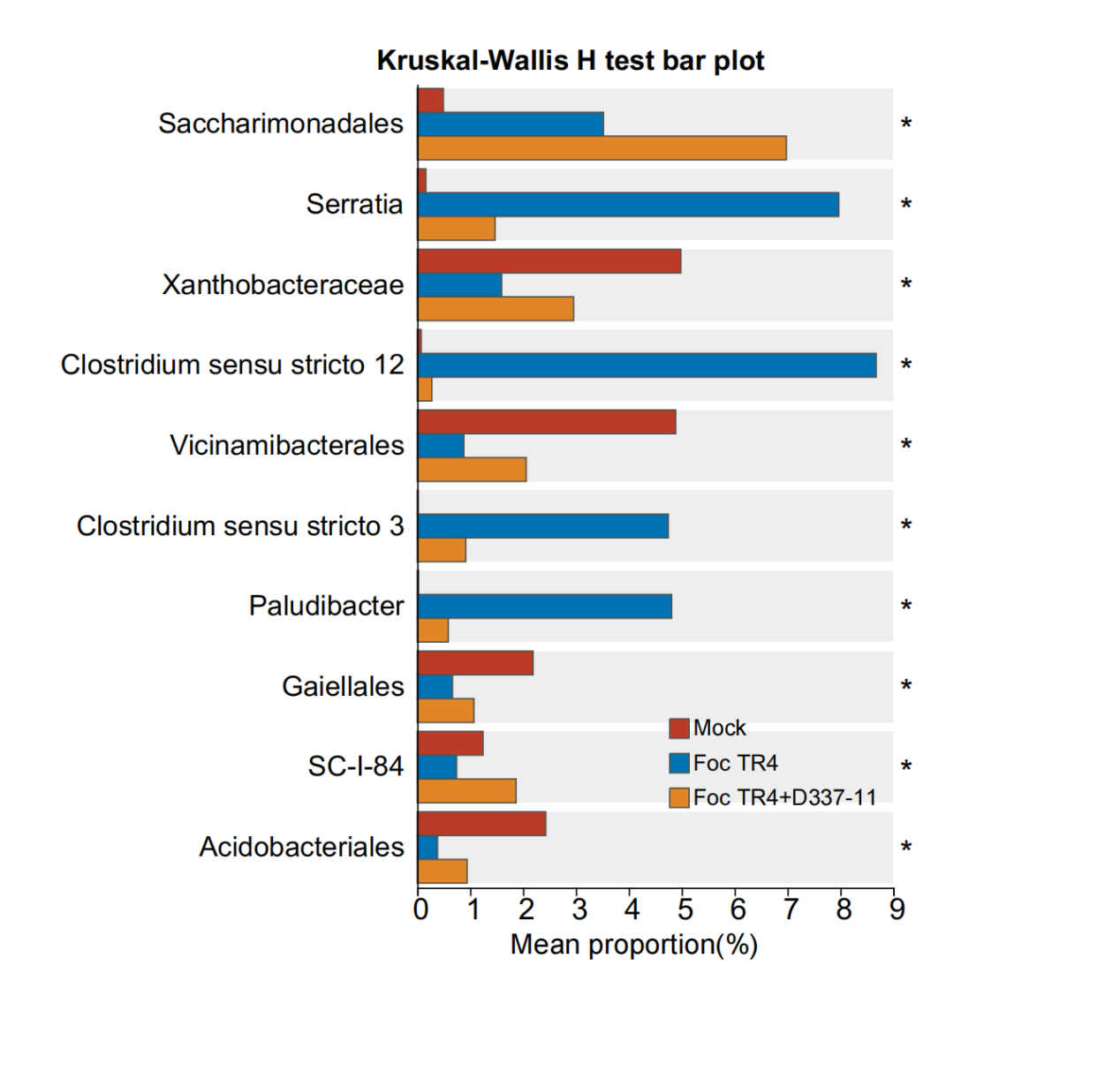


**Figure S4.** The proportion difference of the top 30 genus in abundance between different treatments


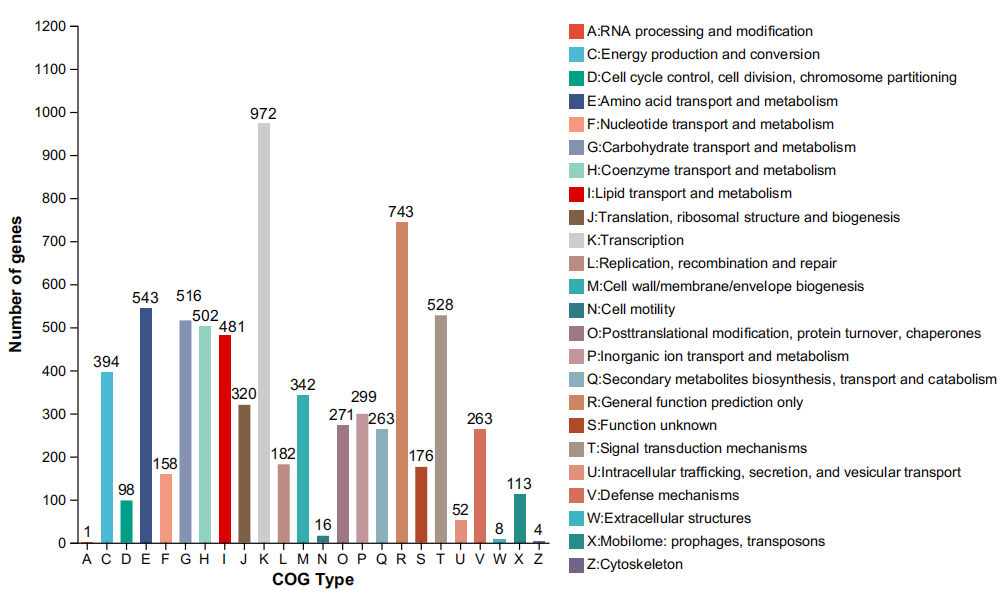


B

A


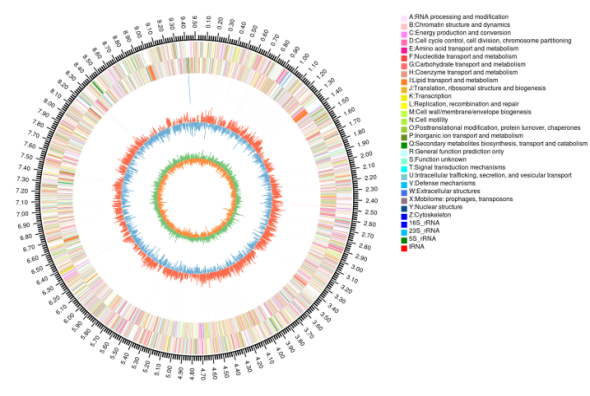

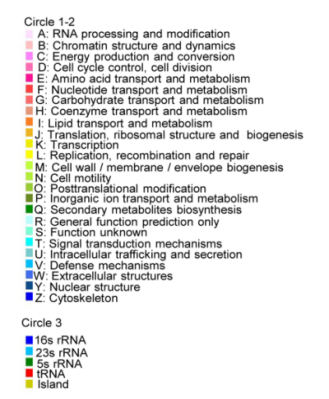


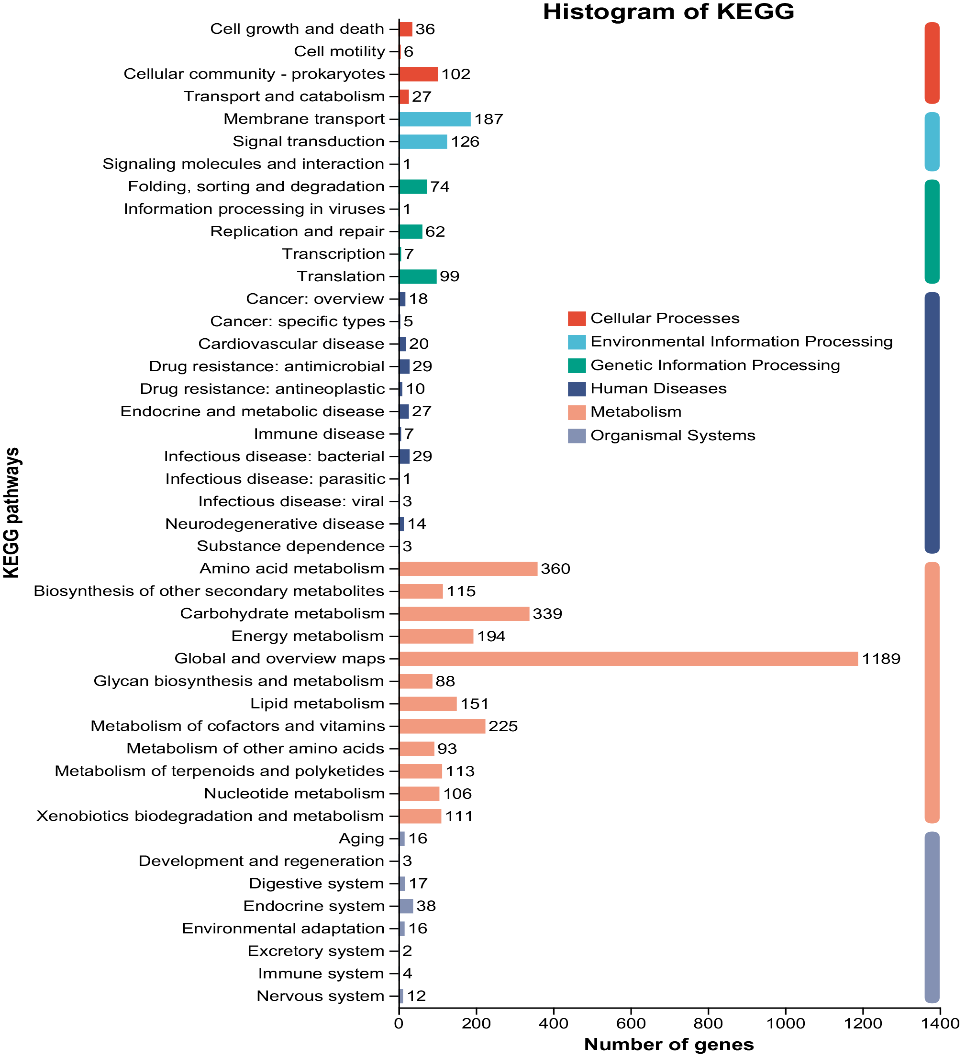


C

**Figure S5.** Genome analysis of *Streptomyces* sp. D337-1. (A) Circular map of strain 2-6 chromosome. (B) COG functional annotation of *Streptomyces* sp. D337-11. (C) Statistical diagram of *Streptomyces* sp. D337-11 on KEGG functional annotation.

A

B

**Figure S6.** (A) Prediction of [secondary](javascript:;) [metabolite](javascript:;)s by alignment with the online antiSMASH v4.2.0 software. (B) Comparative analysis of Region 52.1 in the genome of *Streptomyces* sp. D337-11.


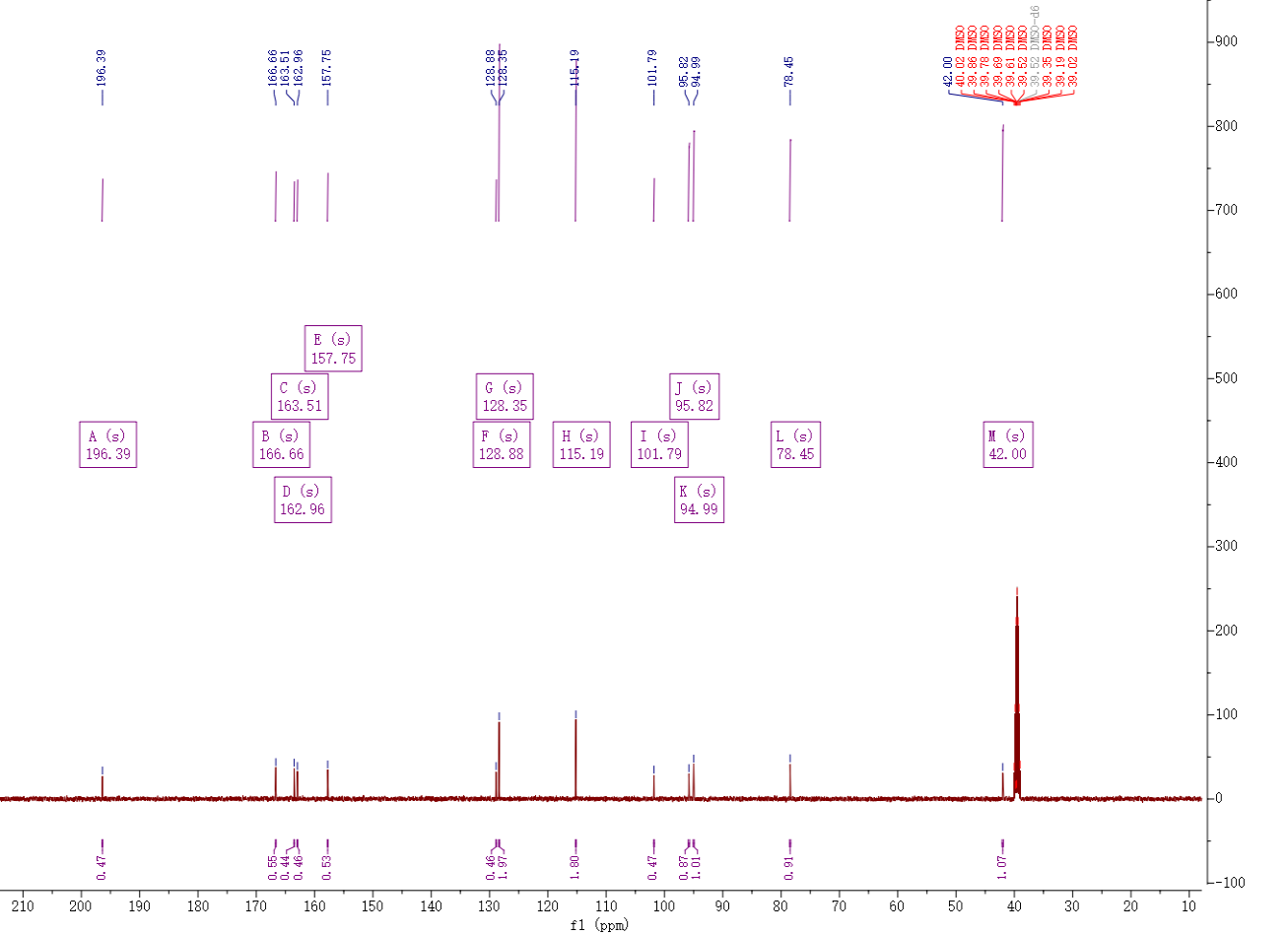


**Figure S7.** ^13^C NMR spectrum of naringenin in (CD_3) 2_SO (125 MHz).

**
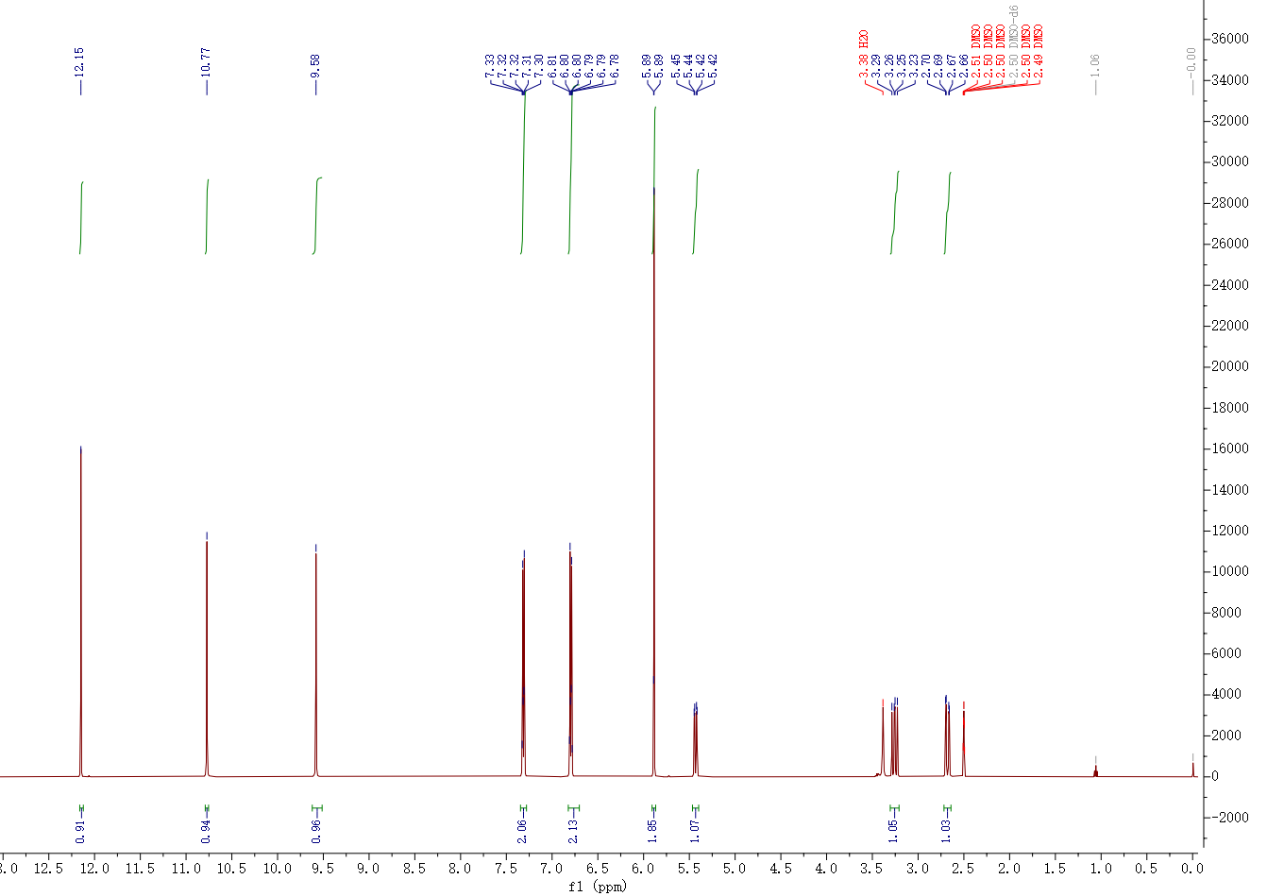
**

**Figure S8.** ^1^H NMR spectrum of naringenin in (CD_3) 2_SO (500 MHz).


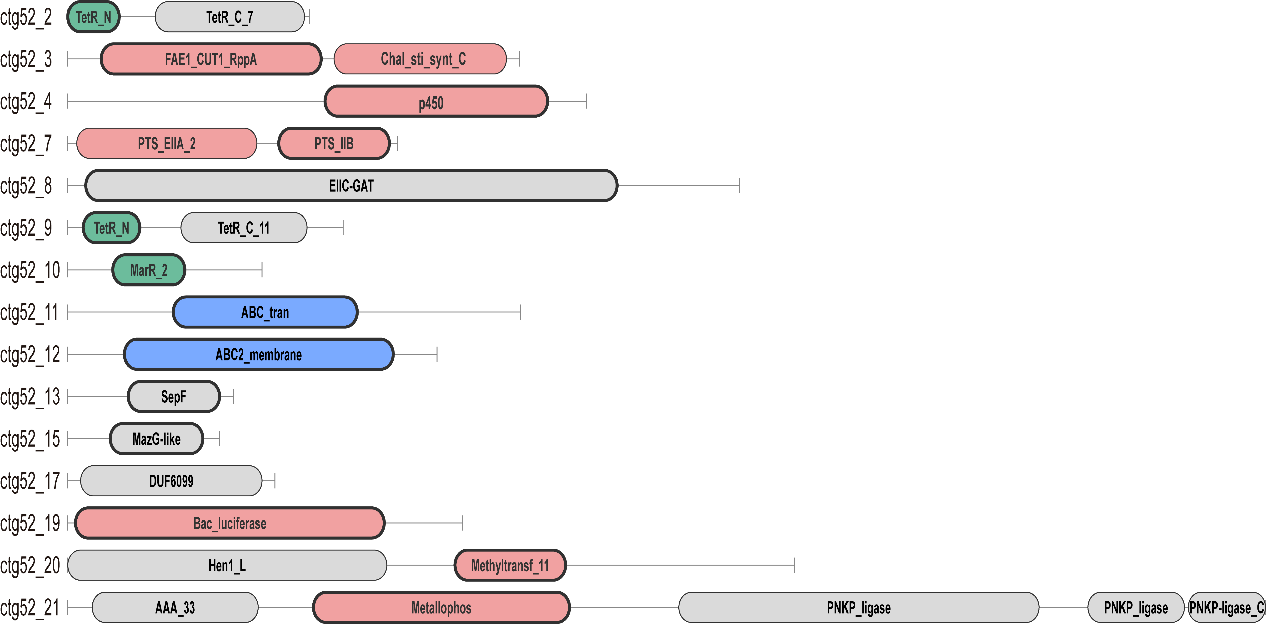


**Figure S9**. Pfam of naringenin in *Streptomyces* sp. D337-11


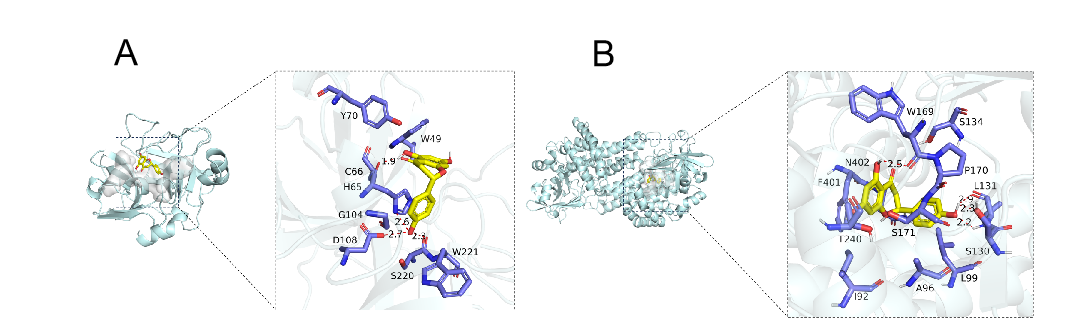


**Figure S10** (A) 3D diagram of binding of Naringenin to the Trypsin Protein . (B) 3D diagram of binding of Naringenin to the Nitroalkane oxidase Protein


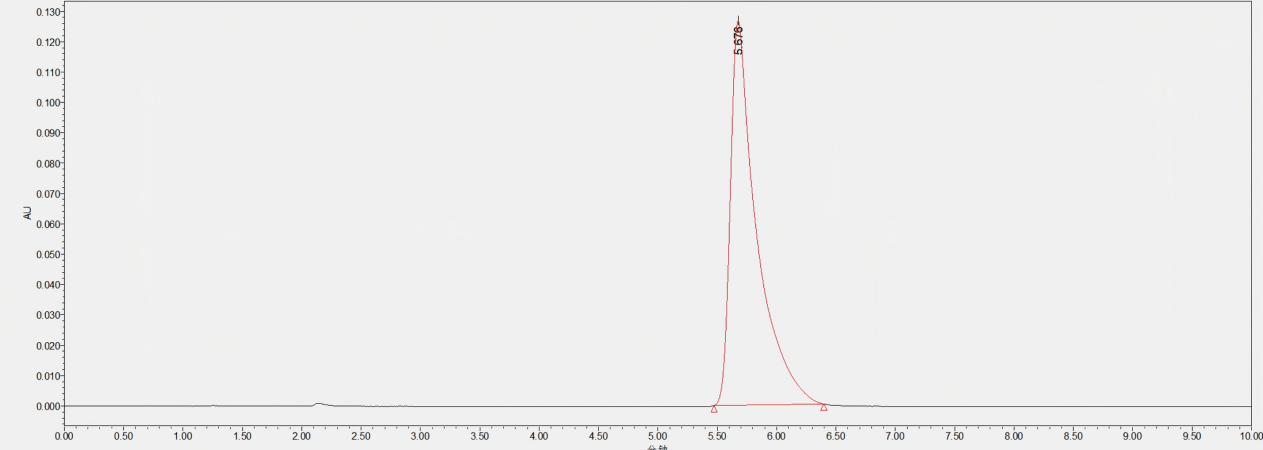
**Figure S11** HPLC analysis of the purified compound of Naringenin

**Table S1**. Actinomycetes strains with strong antagonistic effects against *Foc* TR4

| Strain NO. | Inhibition rate (%) | Strain NO. | Inhibition rate (%) |
| --- | --- | --- | --- |
| D040-14 | 65.30±0.71 | D048-27 | 71.18±0.57 |
| D040-65 | 61.56±0.83 | D048-41 | 64.12±0.46 |
| D047-1 | 61.31±0.95 | D337-11 | 74.41±1.04 |
| D043-12 | 67.82±1.44 | D052-19 | 62.78±0.30 |
| D043-15 | 61.76±0.55 | D052-31 | 60.88±0.34 |
| D043-16 | 61.76±0.65 | D052-46 | 69.37±0.18 |
| D043-18 | 62.35±1.03 | D052-50 | 60.96±0.62 |
| D043-22 | 65.93±0.33 | D052-57 | 62.76±0.11 |
| D043-25 | 69.70±0.04 | D052-83 | 62.60±0.42 |
| D043-5 | 60.00±0.02 | D092-46 | 66.67±1.1 |
| D047-17 | 64.29±0.22 | D092-58 | 70.20±0.57 |
| D047-24 | 61.90±0.34 | D092-63 | 62.75±0.64 |

**Table S2.** Growth characteristics of strain D337-11 on different solid culture media

|  | Aerial mycelium | Vegetative mycelium | Soluble pigment | Growth | Colony |
| --- | --- | --- | --- | --- | --- |
| ISP2 | Deep yellow | White | None | +++ |  |
| ISP3 | Snow | Light yellow | None | ++ |  |
| ISP4 | Light yellow | White | None | ++ |  |
| ISP5 | Light yellow | White | None | +++ |  |
| ISP6 | yellow | White | None | +++ |  |
| ISP7 | yellow | White | None | +++ |  |
| GS no.1 | Azure | Light yellow | None | + |  |

+ good growth; ++ better growth; +++ best growth

**Table S3.** Physiological and biochemical characteristics of strain D337-11

| Characteristics | Results |
| --- | --- |
| PH range for growth | 4-9 |
| NaCl tolerance for growth (%) | 1-3 |
| Gelatin liquefaction | + |
| H2S production | - |
| Nitrate reduction | + |
| Starch hydrolysis | + |
| Cellulose hydrolysis | + |
| Urease production | - |
| Esterase production | + |
| Solubilizing capacity | + |
| Nitrogen fixing capacity | + |
| Potassium capacity | - |

+ Positive reaction; − Negative reaction

**Table S4.** Carbon and nitrogen utilization characteristics of strain D337-11

| Carbon Source Utilizition | | Nitrogen Source Utilization | |
| --- | --- | --- | --- |
| Carbon Source | Result | Nitrogen Source | Result |
| D-Fructose | + | L-arginine | + |
| D-Galactose | + | tyrosine | + |
| D-Mannose | + | Glycine | + |
| D-Sorbitol | + | Methionine | + |
| D-Trehalose | + | Tryptophan | + |
| D-Xylose | + | L (+)-cysteine | + |
| L-Arabinose | + | Phenylalanine | + |
| Melitose | + | Valine | + |
| D-Mannitol | + | Histidine | + |
| Inositol | + | Glutamate | - |
| Melezitose | + | aspartic acid | - |
| Rhamnose | + | creatine anhydrous | + |
| Ribose | + |  |  |
| Maltose | + |  |  |
| Sucrose | + |  |  |
| Glucose | + |  |  |

**Table S5. Antibiotic** test of antagonistic strain D337-11

| Number | Types of Antibiotics | Content (μg/slice) | Result |
| --- | --- | --- | --- |
| 1 | Fluoxetine | 300 | R |
| 2 | doxycycline | 30 | R |
| 3 | Imipenem | 10 | S |
| 4 | Levofloxacin | 5 | R |
| 5 | Clindamycin | 2 | R |
| 6 | Chloramphenicol | 30 | R |
| 7 | Paediatric Compound Sulfamethoxazole Tablets | 25 | S |
| 8 | Polymyxin B | 300IU | S |
| 9 | Vancomycin | 30 | R |
| 10 | Lincomycin | 2 | S |
| 11 | Ciprofloxacin | 5 | R |
| 12 | Norfloxacin | 10 | R |
| 13 | Azithromycin | 15 | R |
| 14 | Erythromycin | 15 | R |
| 15 | Minocycline | 30 | R |
| 16 | Tetracycline | 30 | S |
| 17 | Streptomycin | 10 | R |
| 18 | Kanamycin | 30 | R |
| 19 | Gentamicin | 10 | R |
| 20 | Amikacin | 30 | R |
| 21 | Cefepime | 75 | S |
| 22 | Piperacillin-tazobactam | 100 | S |
| 23 | Ampicillin | 100 | S |
| 24 | Phenoxymethylpenicillin | 1 | S |
| 25 | Penicillin | 10 | S |

**\**

**Table S6.** Prediction and functional annotated statistics of secondary metabolites in *Streptomyces* sp. D337-11

| **Gene cluster type** | **Gene cluster number** | **Gene dosage** | **Function** | **References** |
| --- | --- | --- | --- | --- |
| butyrolactone | 1 | 9 | oxidation resistance | Yang et al., 2023 |
| CDPS | 3 | 54 | not reported |  |
| hglE-KS | 2 | 55 | not reported |  |
| ectoine | 1 | 9 | pervasive quality | Xu et al., 2023 |
| -class-i | 1 | 18 | antimicrobial activity | Long et al., 2024 |
| lanthipeptide-class-iii | 1 | 46 |  |  |
| lassopeptide | 1 | 22 | antimicrobial activity | Li et al., 2022 |
| linaridin | 2 | 39 | antiviral activity | Lin et al., 2023 |
| RiPP-like | 2 | 18 |  |  |
| NAPAA | 2 | 61 | not reported |  |
| NRPS | 7 | 96 | antimicrobial activity | Jiang et al., 2023 |
| NRPS-like | 3 | 87 |  |  |
| siderophore | 2 | 16 | antifungal activity | Cunrath et al., 2020 |
| T1PKS | 18 | 274 | antimicrobial activity | Xie et al., 2024 |
| T2PKS | 1 | 77 |  |  |
| T3PKS | 1 | 18 |  |  |
| transAT-PKS | 1 | 31 |  |  |
| terpene | 7 | 199 | antimicrobial activity | Paddon et al., 2013 |
| thiopeptide | 1 | 46 | antibacterial activity | Bai et al., 2022 |
| other | 1 | 53 |  |  |
| total | 58 | 1228 |  |  |

**Table S7.** Significant differential metabolites of *Streptomyces* sp. D337-11 (Top 35)

| Metabolite | RT | LM | VIP | FC (T/CK) | P-value | FDR | m/z | Activity |
| --- | --- | --- | --- | --- | --- | --- | --- | --- |
| **7-Epijasmonic acid** | **2.4742** | **pos** | **2.6395** | **62.7439** | 1.33E-25 | **8.24E-23** | **228.1593994** | **No activity** |
| 10-Hydroxydecanoic acid | 2.596966667 | pos | 2.6206 | 27.7939 | 1.59E-22 | 2.52E-20 | 230.1750358 | Antibacterial activity |
| Naringenin | 5.18455 | neg | 2.5778 | 18.2675 | 2.09E-22 | 4.35E-20 | 271.0611957 | Antibacterial activity |
| 3alpha-Hydroxyoreadone | 5.5167 | pos | 2.5169 | 11.4834 | 1.44E-11 | 6.06E-11 | 235.132733 | No activity |
| Phaseic acid | 4.126266667 | pos | 2.7502 | 10.1038 | 3.08E-07 | 5.69E-07 | 298.1646355 | No activity |
| 5-((6-((Aminomethyl) amino)-1-oxohexyl) amino) pentanoic acid | 0.59745 | pos | 2.6682 | 8.7992 | 5.09E-26 | 3.60E-23 | 290.218275 | No activity |
| Pentalenolactone O | 5.970433333 | neg | 2.6855 | 8.2976 | 6.38E-07 | 1.10E-06 | 587.2135336 | No activity |
| Beta-Guanidinopropionic acid | 4.272633333 | neg | 2.4451 | 7.431 | 1.56E-10 | 4.85E-10 | 261.1341725 | No activity |
| Ribalinium | 5.4801 | neg | 2.6768 | 5.0665 | 5.74E-12 | 2.41E-11 | 311.1135586 | No activity |
| 3-Hydroxyphenylacetic Acid | 4.608033333 | pos | 2.1774 | 4.8406 | 3.95E-07 | 7.15E-07 | 135.0440787 | Antibacterial activity |
| Metoprolol Acid | 5.0008 | neg | 2.6253 | 4.5522 | 1.38E-09 | 3.60E-09 | 266.1396766 | No activity |
| Lincomycin B | 5.46375 | pos | 2.5323 | 4.5035 | 1.35E-10 | 4.55E-10 | 410.2281325 | Antibacterial activity |
| Galactosyl 4-hydroxyproline | 1.875566667 | neg | 2.4496 | 4.2119 | 2.07E-12 | 9.61E-12 | 585.2151084 | No activity |
| 16,16-Dimethyl Prostaglandin E1 | 4.3286 | neg | 2.3647 | 4.1725 | 2.96E-05 | 4.26E-05 | 427.2672207 | No activity |
| Coumaryl acetate | 5.5167 | pos | 2.3377 | 4.1427 | 1.02E-07 | 2.01E-07 | 175.0753614 | No activity |
| Moringyne | 5.13525 | pos | 2.3584 | 4.1176 | 2.11E-11 | 8.63E-11 | 277.1070633 | No activity |
| 1,2-Dimethyl-4-(6-methyl-4-heptenyl)-1,3-cyclohexadiene | 6.1196 | pos | 2.4861 | 4.0028 | 9.36E-09 | 2.20E-08 | 260.2370978 | No activity |
| PS (22:6 (4Z,7Z,11E,13Z,15E,19Z)-2OH (10S,17)/22:5 (4Z,7Z,10Z,13Z,16Z)) | 5.87645 | neg | 2.408 | 3.9717 | 1.41E-22 | 3.16E-20 | 958.5020553 | No activity |
| Octadecyl fumarate | 6.151483333 | neg | 2.2015 | 3.894 | 1.94E-10 | 5.90E-10 | 405.2391825 | No activity |
| 5'-O-beta-D-Glucosylpyridoxine | 3.864316667 | neg | 2.1782 | 3.2146 | 2.98E-08 | 6.15E-08 | 312.1090188 | No activity |
| Methyl isoeugenol | 4.809866667 | pos | 2.1266 | 3.1538 | 1.98E-14 | 1.83E-13 | 143.0855146 | No activity |
| [(2S,3R)-2-Amino-3-hydroxybutanoyl] (2S)-pyrrolidine-2-carboxylate | 4.552833333 | neg | 2.2468 | 3.1491 | 2.83E-10 | 8.31E-10 | 647.3262091 | No activity |
| Primidone | 4.440866667 | neg | 2.1306 | 3.008 | 6.22E-09 | 1.44E-08 | 263.103673 | No activity |
| Cadabicine | 4.86485 | neg | 2.3698 | 2.954 | 1.21E-07 | 2.28E-07 | 456.1875682 | No activity |
| Bisphenol A | 5.88205 | pos | 2.2896 | 2.9172 | 1.05E-06 | 1.80E-06 | 246.1487249 | No activity |
| Deoxynivalenol | 4.483583333 | pos | 2.074 | 2.8958 | 3.08E-20 | 2.07E-18 | 338.1573945 | No activity |
| Arginylthreonine | 5.6311 | neg | 2.2839 | 2.8369 | 4.45E-07 | 7.82E-07 | 310.1295579 | No activity |
| 4-Hydroxycinnamic acid | 4.499066667 | pos | 2.2408 | 2.7406 | 7.65E-28 | 2.62E-24 | 346.1315045 | No activity |
| Arginylproline | 4.3286 | neg | 2.3579 | 2.728 | 1.13E-12 | 5.53E-12 | 308.1139066 | No activity |
| 1,11-Undecanedicarboxylic acid | 4.272633333 | neg | 2.1701 | 2.7052 | 1.49E-14 | 1.18E-13 | 289.1656315 | No activity |
| DG (8:0/i-12:0/0:0) | 6.2466 | neg | 2.0354 | 2.6951 | 7.63E-20 | 4.12E-18 | 435.2864749 | No activity |
| Methyleugenol | 5.15815 | pos | 2.1386 | 2.6769 | 2.14E-16 | 3.97E-15 | 143.0854953 | Antibacterial activity |
| 13 (S)-Hydroperoxylinolenic acid | 4.499066667 | pos | 2.2827 | 2.6745 | 1.19E-07 | 2.32E-07 | 328.2478772 | No activity |
| (3-Methylphenyl) methyl acetate | 5.74985 | neg | 2.2302 | 2.6516 | 5.24E-27 | 1.47E-23 | 163.0757063 | No activity |

Table S8 ^1^H-NMR and ^13^C-NMR data for naringenin in (CD_3) 2_SO (*J* in Hz).

| **Position** | ***δ*_H_ (mult, *J* in Hz)** | ***δ*_C_** |
| --- | --- | --- |
| 2 | 5.42 (dd, 13.2, 3.0) | 78.45, d |
| 3 | 2.69 (dd, 17.2, 3.0) | 42, t |
|  | 3.25 (dd, 17.2, 13.2) |  |
| 4 |  | 196.39, s |
| 5 |  | 163.51, s |
| 6 | 5.90 (d, 2.0) | 94.99, d |
| 7 |  | 166.66, s |
| 8 | 5.89 (d, 2.0) | 95.82, d |
| 9 |  | 162.96, s |
| 10 |  | 101.79, s |
| 1´ |  | 128.88, s |
| 2´ | 7.32 (dd, 7.2, 1.8) | 128.35, d |
| 3´ | 6.83 (dd, 6.6, 2.4) | 115.19, d |
| 4´ |  | 157.75, s |
| 5´ | 6.83 (dd, 7.2, 1.8) | 115.19, d |
| 6´ | 7.32 (dd, 7.2, 1.8) | 128.35, d |
